# Supplementary material for: The inwardly rectifying K+ channel KIR7.1 controls uterine excitability throughout pregnancy
Source: EMBO Mol Med. 2014 Jul 23;6(9):1161–74. doi: 10.15252/emmm.201403944 (PMC4197863; doi:10.15252/emmm.201403944)
Supplement: Supplementary file 1 — Supplementary Figure S1 [file emmm0006-1161-SD1.pdf]

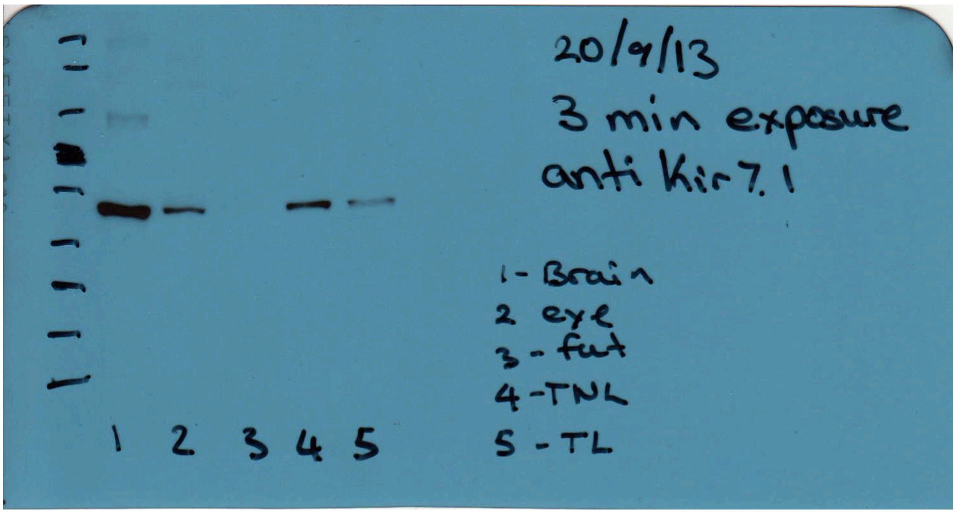

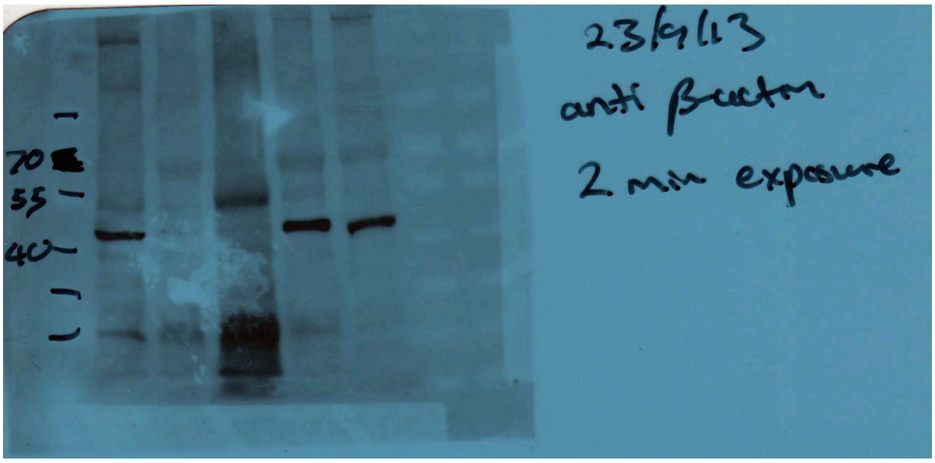

Figure S1.

Full scan of immune-blot depicted in Figure 1c. 40µg total protein was loaded per lane. Adult human eye and adult mouse brain act as positive control and adult mouse adipose tissue acts as negative control. Blot was re-probed for β-actin to ensure equal loading of myometrial samples. NB. β-actin was more highly expressed in brain and myometrium than eye and adipose tissue.
