# Supplementary material for: The inwardly rectifying K+ channel KIR7.1 controls uterine excitability throughout pregnancy
Source: EMBO Mol Med. 2014 Jul 23;6(9):1161–74. doi: 10.15252/emmm.201403944 (PMC4197863; doi:10.15252/emmm.201403944)
Supplement: Supplementary file 2 — Supplementary Figure S2 [file emmm0006-1161-SD2.pdf]

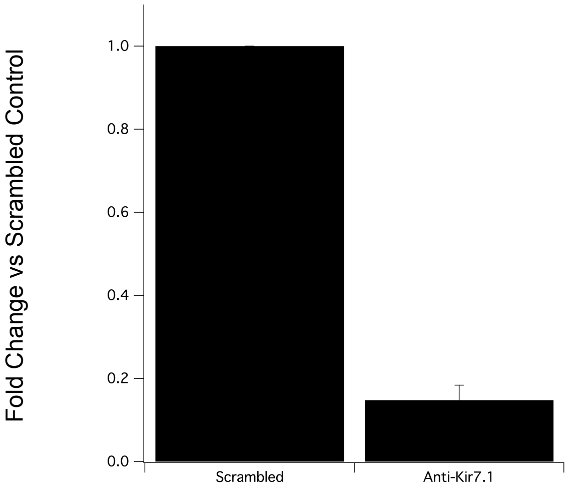

Figure S2.

Control qRT-PCR (n=3 biological replicates assayed in triplicate) of relative (fold change vs Scrambled control) Kir7.1 mRNA levels in isolated myometrium infected with Anti-Kir7.1 mRNA. > 80% Knockdown was achieved.
