# Supplementary material for: The inwardly rectifying K+ channel KIR7.1 controls uterine excitability throughout pregnancy
Source: EMBO Mol Med. 2014 Jul 23;6(9):1161–74. doi: 10.15252/emmm.201403944 (PMC4197863; doi:10.15252/emmm.201403944)
Supplement: Supplementary file 3 — Supplementary Figure S3 [file emmm0006-1161-SD3.pdf]

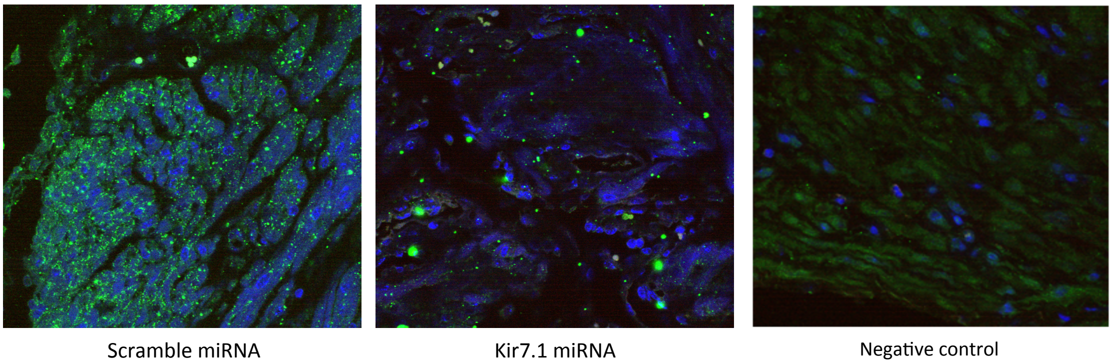

Figure S3. Immunofluorescence performed on GD15 transfected uteri using Kir7.1 antibody (green) to label the Kir7.1 channel to confirm protein knockdown *in vivo* while blue marks nuclear staining (n=4). Negative control was as scramble miRNA but without primary antibody to Kir7.1. Uterus was transduced at P10 for both scramble and Kir7.1 miRNA (60x).
