# Supplementary material for: The inwardly rectifying K+ channel KIR7.1 controls uterine excitability throughout pregnancy
Source: EMBO Mol Med. 2014 Jul 23;6(9):1161–74. doi: 10.15252/emmm.201403944 (PMC4197863; doi:10.15252/emmm.201403944)
Supplement: Supplementary file 4 — Supplementary Figure S4 [file emmm0006-1161-SD4.pdf]

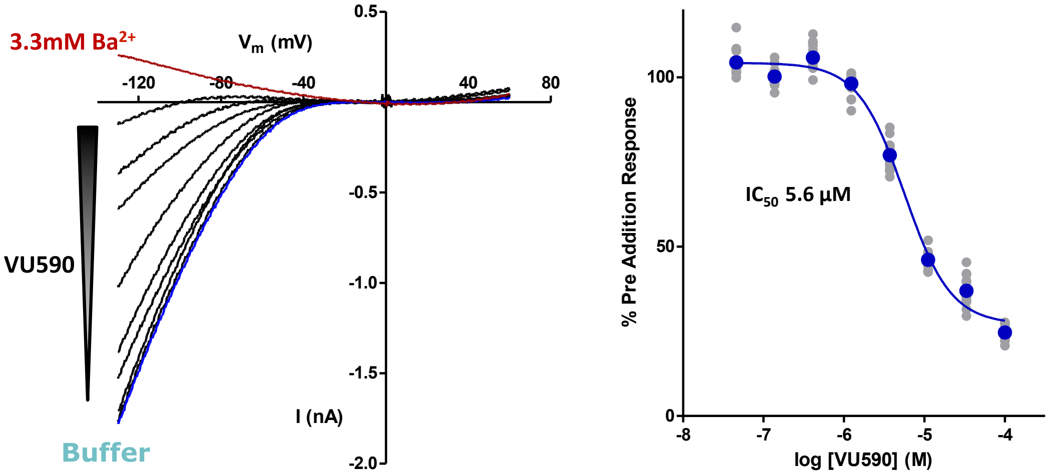

Figure S4.

Population patch-clamp current voltage relation of hKir7.1 current in CHO cells and inhibition by VU590 + 3.3mM Ba^{2+}
