# Supplementary material for: The inwardly rectifying K+ channel KIR7.1 controls uterine excitability throughout pregnancy
Source: EMBO Mol Med. 2014 Jul 23;6(9):1161–74. doi: 10.15252/emmm.201403944 (PMC4197863; doi:10.15252/emmm.201403944)
Supplement: Supplementary file 5 — Supplementary Figure S5 [file emmm0006-1161-SD5.pdf]

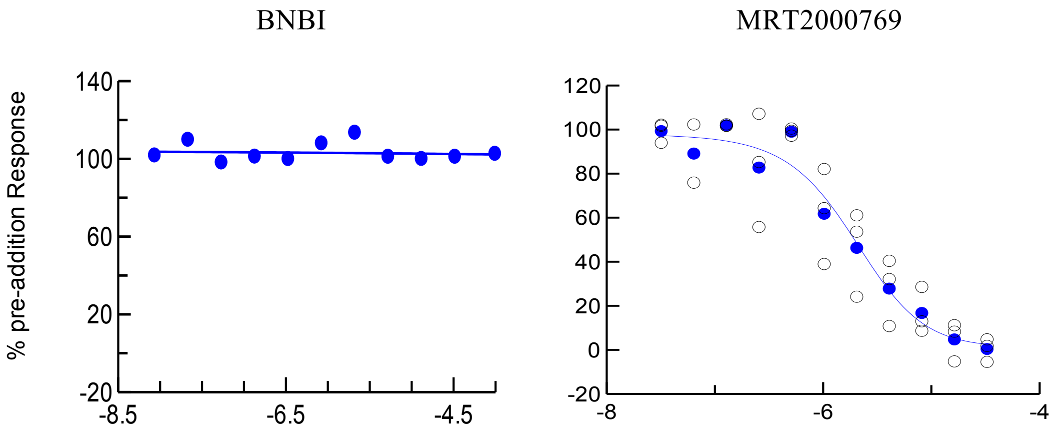

Figure S5.

Log[Drug] inhibition curve determined from population patch-clamp of hKir7.1 current in CHO cells (see supplementary methods for detail). BNBI a known inhibitor of Kir1.1 demonstrated no inhibition of Kir7.1. MRT2000769 by contrast is a potent inhibitor of Kir7.1 IC_{50} 2.0μM [1.1-3.5] (mean, 95%CI).
