# Supplementary material for: The inwardly rectifying K+ channel KIR7.1 controls uterine excitability throughout pregnancy
Source: EMBO Mol Med. 2014 Jul 23;6(9):1161–74. doi: 10.15252/emmm.201403944 (PMC4197863; doi:10.15252/emmm.201403944)
Supplement: Supplementary file 6 — Supplementary Figure S6 [file emmm0006-1161-SD6.pdf]

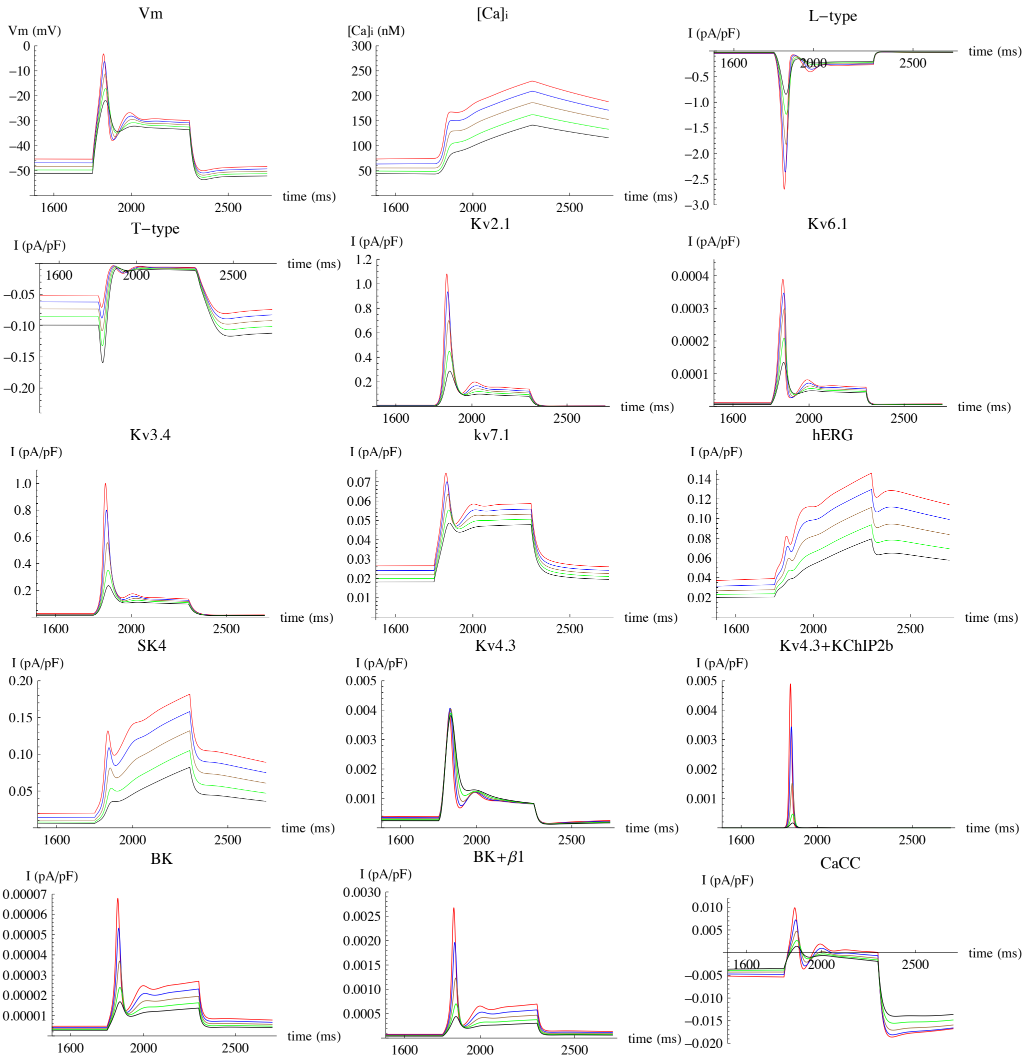

Figure S6: Effect of increasing Kir7.1 densities.

A free running simulation of the effect of increasing Kir7.1 densities on the myometrial AP waveform. Time-dependent effect of increasing Kir7.1 densities (red=0 channels/pF, blue=300 channels/pF, brown=600 channels/pF, green=900 channels/pF and black=1200 channels/pF) is depicted on V (mV), [Ca2+]i (nM), and individual conductances (pA/pF) included in the simulation. Increasing density of Kir7.1 within physiologically realistic values, hyperpolarises RMP, whilst decreasing membrane excitability during depolarising excursions in V leading to decreasing calcium entry. Changing the channel density of Kir7.1 has a disproportionate effect on hERG, Kv7.1, SK4 and BK.
