# Supplementary material for: The inwardly rectifying K+ channel KIR7.1 controls uterine excitability throughout pregnancy
Source: EMBO Mol Med. 2014 Jul 23;6(9):1161–74. doi: 10.15252/emmm.201403944 (PMC4197863; doi:10.15252/emmm.201403944)
Supplement: Supplementary file 7 — Supplementary Table S1 [file emmm0006-1161-SD7.pdf]

Table S1.

The comparison of K+ channels gene expression in multiple uterine cell types and myometrial cells

Gene (Channel)	Whole myometrium (ΔCT) (n = 7)	LCM myometrium (ΔCT) (n = 5)	Fold difference (ΔΔCT) Whole vs LCM	P
**KCNJ13 (Kir7.1)**	22.57 ± 0.17	19.27 ± 1.73	**8.12 (2.71 - 24.3)**	**0.01**
**KCNH2B (Kv11.1)**	21.68 ± 0.19	18.20 ± 2.54	**6.84 (1.69 - 27.8)**	**0.05**
**KCNAB3 (AKR6A9, KCNA3.1B, KV-BETA-3)**	23.79 ± 0.32	21.18 ± 1.14	**5.99 (2.75 - 13.1)**	**0.01**
**KCNG1 (Kv6.1)**	20.50 ± 0.13	19.03 ± 1.41	**2.53 (1.04 - 6.14)**	**0.03**
**KCNMB1 (Slo-Beta)**	15.38 ± 0.28	13.96 ± 0.92	**2.42 (1.37 – 4.30)**	**0.01**
**KCND3 (Kv4.3)**	17.88 ± 0.10	16.79 ± 0.94	**1.99 (1.10 - 3.62)**	**0.04**
**KCNMA1 (Kca1.1, BKca, Maxi K)**	14.92 ± 0.27	13.93 ± 0.46	**1.83 (1.38 – 2.43)**	**0.01**
**KCNB1 (Kv2.1)**	16.19 ± 0.14	17.03 ± 0.69	**0.58 (0.37 - 0.91)**	**0.03**
**KCNQ1 (Kv7.1)**	20.10 ± 0.24	21.48 ± 0.90	**0.39 (0.21 - 0.72)**	**0.03**
**KCND2 (Kv4.2)**	21.00 ± 0.13	23.81 ± 1.74	**0.16 (0.05 - 0.49)**	**0.01**

Table S1 depicts ∂Ct (mean±s.e.) and fold difference (whole vs LCM; ∂∂Ct whole vs LCM (mean, 95% C.I.)) of K^+ channel genes differentially expressed between whole myometrial biopsy and laser captured smooth muscle. Black depicts genes significantly (*P*<0.05, Two tailed Student's T-Test) enriched in laser captured smooth muscle cells. Red depicts genes significantly enriched in non-myometrial smooth muscle in whole myometrial biopsy. KCNJ13 was eightfold higher in laser-captured samples when compared to whole tissue indicating enrichment in myometrial smooth muscle cells.
