# Supplementary material for: The inwardly rectifying K+ channel KIR7.1 controls uterine excitability throughout pregnancy
Source: EMBO Mol Med. 2014 Jul 23;6(9):1161–74. doi: 10.15252/emmm.201403944 (PMC4197863; doi:10.15252/emmm.201403944)
Supplement: Supplementary file 8 — Supplementary Methods [file emmm0006-1161-SD8.pdf]

PCR primer design and qRT-PCR. Genomic DNA and mRNA sequences were downloaded from NCBI LocusLink at http://www.ncbi.nlm.nih.gov/sites/entrez?db=gene for all known potassium channels in the Human Genome (Table M1). For each gene of interest PCR primer pairs were designed to span the 3′-most intron and to generate an amplicon that was shared by all known splice variants. Primer characteristics, such as melting temperature (Tm), GC content, secondary structure formation and primer-end self complementarity, were evaluated and secondary structure minimized for each amplicon. Selected primers were validated initiatially *in silico* using NCBI BLAST and subsequently validated empirically on a control whole body cDNA library. Before running qRT-PCR experiments, the efficiency of amplification was determined for each primer set by using 2-fold serial dilutions of input cDNA. All primer sets exhibited an efficiency > 90. All PCR products exhibited a single peak as determined by melt-curve analysis and produced and correct products when sequenced. Data was collected using the 7500 Real-Time PCR System (Applied Biosystems) and SensiMix Plus SYBR (Quantace) as fluorescent reporter. Relative quantification was performed using the comparative threshold (CT) method after determining the CT values for reference (18SrRNA) and target genes in each sample sets according to the $2^{-\Delta\Delta Ct}$ method (Pfaffl, 2006) as described by the manufacturer (Applied Biosystems; User Bulletin 2). Changes in mRNA expression level were calculated after normalization to 18SrRNA.

$\Delta Ct_{Total} = Ct\_Mean_{Total} - Ct\_Mean_{18SrRNA}$

$\Delta Ct_{LCM} = Ct\_Mean_{LCM} - Ct\_Mean_{18SrRNA}$

$\Delta\Delta Ct = \Delta Ct_{Total} - \Delta Ct_{LCM}$

Gene expression level = $(1+\text{Efficiency})^{-\Delta\Delta Ct}$

Table M1.

PCR primers designed in the current study

Gene	Also known as	Working Primers (5' - 3')	Size (bp)	Tm
**Human Potassium Channels**				
KCNK1	TWIK-1, DPK; HOHO; TWIK1; K2p1.1;	GTCCTGGAGGATGACTGGAA<br>CACCTGATCCTCGTCCTTGT	261	82.0
KCNK2	TREK; TPKC1; TREK1; K2p2.1; TREK-1; hTREK-1c; hTREK-1e; MGC126742; MGC126744	CCGTTAGGAAACACCTCCAA<br>CTTTGGCAATTCCTTTTCCA	229	80.4
KCNK3	TASK-1; OAT1; TASK; TBAK1; K2p3.1	CCTTCTACTTCGCCATCACC<br>GAACATGACGAGCGTGAGC	128	87.0
KCNK4	TRAAK; K2p4.1; TRAAK1	GTGCCACCGGAGCTAGTAAG<br>AGGATCCAGAACCACACCAG	236	87.0
KCNK5	TASK2; K2p5.1; TASK-2; FLJ11035	TTCATCACCATCTCCACCAT<br>CAGCCCCAAGTAGATCCAGA	114	84.4
KCNK6	TOSS; KCNK8; TWIK2; K2p6.1; TWIK-2; FLJ12282	CCCTCTACAAGGTGCTGGTC<br>GTCCTCATCCGCATTGAAAC	158	87.3
KCNK7	TWIK3; K2p7.1; MGC118782; MGC118784	CCTCACCACCACAGGTTATG<br>ACCAGCAGTCCCAGTGCAA	228	89.3
KCNK9	KT3.2; TASK3; K2p9.1; TASK-3; MGC138268; MGC138270	CTACTTTGCGATCACGGTCA<br>GGAGAAGAAGCCCACAGTCA	247	88.0
KCNK12	THIK2; THIK-2	CACCGTGGTGTCAACCATAG<br>GCTCCAGGAAGAGGTTGAAG	128	85.9
KCNK13	THIK1; THIK-1; K2p13.1	CGTTTCCACCATAGGGTTTG<br>CAGGCTGTCCACCTCACACT	241	85.9
KCNK16	TALK1; TALK-1; K2p16.1; MGC133123	CAAGTCACACCCCAGGACTT<br>GCTTGTCCACTCCTTGCTTC	143	83.5
KCNK17	TASK4, TALK2; TALK-2; TASK-4; K2p17.1	GGGATGTCGTCCAAGCATAC<br>AAGGGCAAAGAAGATGCAGA	179	86.0
KCNK18	TRIK; TRESK; TRESK2; K2p18.1; TRESK-2	ATGGAGGTCTCGGGGCAC<br>GTTTTCTGTCTTCCACCACTGTTTCACTGCAGTTCAAG	242	88.0
KCNT1	KCa4.1; bA100C15.2	CGTCTTCAGCATCAGCATGT<br>GAAGACGTGGCTCTCTGTCC	241	87.0
KCNT2	SLICK; KCa4.2; SLO2.1; MGC119610; MGC119611; MGC119612; MGC119613; RP11-58O13.1	CAAAACGACGACAGGGTACA<br>GAAACCTGTAAGCCCCACAA	248	77.0
KCNU1	Slo3; KCNMC1; KCa5.1; Kcnma3	CTGGGGCTTCTGTCCTTACA<br>CGGGTGATCACAAACCTTTT	173	79.1
KCNV1	HNKA; KCNB3; KV2.3; KV8.1	TTGCCATCTTGCCCTTCTAC<br>AGGCCGACTTCTTCGTAACA	202	81.6
KCNV2	Kv8.2; MGC120515	AATGTCTGAGCCCCTAGCTG<br>TCGTCTGTCTGCTCCTCGTA	590	88.6
**voltage-gated channel**				
KCNA1	EA1; MK1; AEMK; HBK1; HUK1; MBK1; RBK1; KV1.1; MGC126782; MGC138385	CCATCATTCCTTATTTCATCAC<br>CTCTTCCCCCTCAGTTTCTC	488	85.6
KCNA2	HK4; MK2; HBK5; NGK1; RBK2; HUKIV; KV1.2; MGC50217	AGAGAATTGGGCCTCCTGAT<br>CCCACTATCTTTCCCCCAAT	197	83.5
KCNA3	MK3; HGK5; HLK3; PCN3; HPCN3; KV1.3; HUKIII	CATCCTGGGAGGAAATGAAA<br>CAGGGTAGGCAACCGAAATA	169	77.9
KCNA4	HK1; HBK4; PCN2; HPCN2; HUKII; KCNA8; KV1.4; KCNA4L	GCTTGAGGGGGAAAAACAAG<br>TACCCAGGTACAATGCCAAA	233	82.2
KCNA5	HK2; HCK1; PCN1; HPCN1; KV1.5; MGC117058; MGC117059	CGAGGATGAGGGCTTCATTA<br>AGGGTCTCCAAGCAGAAGGT	174	85.6
KCNA6	HBK2; KV1.6	CAATGGTGGTGTGAGTCGAG<br>GCGTCTCCACCAGAAAGAAG	185	83.5
KCNA7	HAK6; KV1.7	GTCCAAGCAAGGCTATCTTC<br>AGAGGACCACACCGATGAAG	284	88.1
KCNA10	Kcn1; Kv1.8	GCAGAACATCCCAGGAGAAA<br>GAGACAGGATGGACCCAA	151	80.9
KCNAB1	Shaker-related subfamily, beta member 1 (hKvb3; AKR6A3; KCNA1B; Kvb1.3; hKvBeta3; KV-BETA-1)	TTGCCTGTGGAATCATCTCA<br>CCAGGAGCACAGAACTCACA	231	81.8
KCNAB2	shaker-related subfamily, beta member 2 (AKR6A5; KCNA2B; HKvbeta2; KV-BETA-2; MGC117289; HKvbeta2.1; HKvbeta2.2)	TGTTCCAGCGTGAGAAAGTG<br>GGGATGCCACTGTCGTACTT	124	85.0
KCNAB3	Shaker-related subfamily, beta member 3 (AKR6A9; KCNA3B; KCNA3.1B; KV-BETA-3; MGC116886)	CCTCCATCAAGGGCTACCA<br>GCTGGCTCAGCACCTGTAG	234	87.7
KCNB1	Shab-related subfamily, member 1 (Kv2.1; DRK1; KV2.1; h-DRK1)	CCGCTACCACCAGAAGAAAG<br>ATGGTGGAGAGGACGATGAA	207	85.0
KCNB2	Shab-related subfamily, member 2 (Kv2.2)	CTGGAAGTGTGCGACGACTA<br>GGCAGCAGGACTCCAAGTAG	193	80.7
KCNC1	Shaw-related subfamily, member 1 (Kv3.1; KV4; NGK2; FLJ41162; FLJ42249; FLJ43491; MGC129855)	CCGTACTCGTCCCGCTAC<br>ACGCCCTCGATGTAGGTAAG	206	86.0
KCNC3	Shaw-related subfamily, member 3 (Kv3.3; SCA13; KSHIIID)	GCTCTTCGAGGACCCCTACT<br>TATGGATGAAGCCCTCATGG	119	84.0

KCNC4	Shaw-related subfamily, member 4 (Kv3.4; KSHIIIC; HKSHIIIC; MGC126818)	CGCTCTTCGAGGATCCCTAC<br>TCACGTTGCGGTCGATATTA	132	83.1
KCND1	Shal-related subfamily, member 1 (Kv4.1)	GAGAAGACAACGTGCCATGA<br>TGACTGAGGCAGTGGAGTTG	199	87.7
KCND2	Shal-related subfamily, member 2 (Kv4.2; RK5; KIAA1044; MGC119702; MGC119703)	CTTCGGCTAGCAAGTTCACC<br>TGTCTGCTCGTTGATTCTGG	216	81.8
KCND3	Shal-related subfamily, member 3 (Kv4.3; KCND3L; KCND3S; KSHIVB; MGC142035; MGC142037)	CATGGCCATCATCATCTTTG<br>CTGGTGGTAAATCCGGCTAA	250	84.8
KCNE1	Isk-related family, member 1 (ISK; JLNS; LQT5; MinK; JLNS2; MGC33114)	CGTGCTCAGGAGGAAGAGAC<br>AACTGGCCAGAGAAAGCAGA	167	76.0
KCNE1L	KCNE1-like (KCNE5)	ATTTTCTTGCCTCCCCAGTT<br>AAGTTCCACGGATGAAGCAG	169	80.3
KCNE2	Isk-related family, member 2 (LQT5; LQT6; MIRP1; MGC138292)	TTGTGTGCAACCCAGAAGAG<br>CTTCCAGCGTCTGTGTGAAA	147	82.0
KCNE3	Isk-related family, member 3 (HOKPP; MiRP2; MGC102685; MGC129924; DKFZp781H21101)	GTCTGAGCTTCTACCGAGTCTTCC<br>CTCGTGTTAGATCATAGACACACGG	363	84.4
KCNE4	Isk-related family, member 4 (MIRP3; MGC20353)	ACCCTCTTGGACTGGACGAT<br>GTGCTGTTCAGAGGCTCCAT	109	81.7
KCNF1	subfamily F, IK8; kH1; KCNF; KV5.1; MGC33316	CGACACCATCTTCTCCCTGT<br>ATGCCCTTCTTCATGTGGAC	132	82.1
KCNG1	subfamily G, member 1 (K13; kH2; KCNG; KV6.1; MGC12878)	GTCAACCTCTCCGTCAGCAC<br>AGGAGGAACTCCAGGGAGAA	131	85.5
KCNG2	subfamily G, member 2 (KCNF2; KV6.2)	CGTGTCCGTGTCCTTCGT<br>GGAACTCGAAGGAGAACCAG	158	88.7
KCNG3	subfamily G, member 3 (Interacts Kv2.1); also known as KV6.3; KV10.1	GTGCATCGTGAGGTTCATTG<br>GGCAAGCTTAATCACCCAAA	205	81.0
KCNG4	subfamily G, member 2 (KV6.3; KV6.4; MGC4558; MGC129609)	GTCAGCCTGTGTGTCAGCA<br>CGACACGTAGTATGGGGAGA	222	84.9
KCNH1	subfamily H (eag-related), member 1 (EAG, EAG1, Kv10.1, h-eag)	GGAGTTCCAGACGGTGCAC<br>CCTCATCATCTTGGATCACC	116	82.9
KCNH2	subfamily H (eag-related), member 2 (ERG1, HERG, HERG1, Kv11.1, LQT2, SQT1)	GCCTTCTCAGGAGTGTCCAA<br>CGTCATCTGCCTCTGTAGCA	249	89.3
KCNH2B		ACACCTTCCTGGACACCATC<br>AAGCCGTCGTTGCAGTAGAT	109	84.0
KCNH3	subfamily H (eag-related), member 3 (Bec1, Elk2)	TTCCTTTATGGGCCAGACAC<br>TTCGCTGATGTCCTTGTGAG	201	85.3
KCNH4	subfamily H (eag-related), member 4 (BEC2; ELK1; Kv12.3)	ATTCCCTTTTCCAAGCCTTC<br>GGTCCTGGGGAGAAAGTAGG	137	84.3
KCNH5	subfamily H (eag-related), member 5 (EAG2; H-EAG2; Kv10.2)	GCCTGGCTGGTACTGGATAG<br>CAGTCGTAAGAGACGCACCA	258	80.7
KCNH7	subfamily H (eag-related), member 7 (ERG3; HERG3; Kv11.3; MGC45986)	ATTGCTGGGGTCAGAAGAGA<br>GGTGGCAGCGTTTTCATTAT	166	80.0
KCNH8	subfamily H (eag-related), member 8 (ELK, ELK1, Kv12.1)	CAAAGGAATCCCCCATTTTT<br>TTGACATGAGTTGGCAGAGC	184	79.1
KCNQ1	KQT-like subfamily, member 1 (LQT; RWS; WRS; LQT1; SQT2; ATFB1; JLNS1; KCNA8; KCNA9; Kv1.9; Kv7.1; KVLQT1; FLJ26167)	TTTGCCATCTCCTTCTTTGC<br>CTGGGTGACAGCAGAGTGTG	224	86.6
KCNQ2	KQT-like subfamily, member 2 (EBN; BFNC; EBN1; ENB1; HNSPC; KV7.2; KCNA11; KVEBN1)	CGGCAGAACTCAGAAGAAGC<br>ATGACGTCCATCACGTCGTA	194	88.9
KCNQ3	KQT-like subfamily, member 3 (BFNC2, EBN2, KV7.3)	ATCGGGTTCGCCTTTCTAAT<br>CATGTCTTCGATGGGGAAGT	251	84.4
KCNQ4	KQT-like subfamily, member 4 (DFNA2; KV7.4)	CAAACTCGGGTGGACCAAAT<br>GGTAGTCGGAGGTGATGTCG	268	89.2
KCNQ5	KQT-like subfamily, member 5 (Kv7.5) M current forms heteromer with Q3	CGCTTTCGTTTTTCTCCTTG<br>CGAGCAAACCTCAGTCTTCC	207	81.9
KCNS1	delayed-rectifier, subfamily S, member 1 (Kv 9.1)	GGAGGGAAGGAGGATGAGTC<br>GCAGGGATTGGACCACTTTA	206	82.2
KCNS2	delayed-rectifier, subfamily S, member 2 (Kv 9.2)	GGACGTGTCGGAGGCTAAC<br>TTGCCGGTGTGATAGAAATG	245	89.0
KCNS3	delayed-rectifier, subfamily S, member 3 (Kv 9.3)	CAAATGCCTATGTGCACCAC<br>TGCTTTGGTCAACAGACTGC	271	84.8
**Inwardly-rectifying channel**				
KCNJ1	Subfamily J, member 1 (ROMK; ROMK1; KIR1.1)	TGGACATCTGGACAACGGTA<br>CACACAGGGAGTGTGATTGG	170	81.0
KCNJ2	Subfamily J, member 2 (IRK1; LQT7; SQT3; HHIRK1; KIR2.1; HHBIRK1)	TGTTGGGTTTGACAGTGGAA<br>CCCACAGGATTTCATTTGCT	215	81.6
KCNJ3	Subfamily J, member 3 (KGA; GIRK1; KIR3.1)	GGACGGAAAACTCACGCTTA<br>TCAAGTTGGTCAAGGGGAAG	135	83.9
KCNJ4	Subfamily J, member 4 (HIR; HRK1; IRK3; HIRK2; Kir2.3; MGC142066; MGC142068)	ATCGCGCCTTGGGATGTA<br>GTCCGTGCATGTCCTGAAG	115	88.3
KCNJ5	Subfamily J, member 5 (CIR; GIRK4; KATP1; KIR3.4)	GCTTTTCGAGTGCTTTTTGG<br>GGCCCAGATGCTGATTTTTA	250	82.8
KCNJ6	Subfamily J, member 6 (BIR1; GIRK2; KATP2; KCNJ7; KIR3.2; hiGIRK2; MGC126596)	CGCTGATCATTAGCCATGAA<br>CCAGCTCTTTGGCACTAAGG	285	84.0

KCNJ8	Subfamily J, member 8 (KIR6.1; uKATP-1)	CCATGGGGACATCTATGCTT<br>TCATCATCCTCCCTCCAAAC	155	79.3
KCNJ9	Subfamily J, member 9 (GIRK3; KIR3.3)	TGGAGGGCTTGGTAAAACAC<br>CCCATGTCCTGAAACGACTT	183	82.8
KCNJ10	Subfamily J, member 10 (KIR1.2; KIR4.1; BIRK-10; KCNJ13-PEN)	AGCTCCGGGTTTAAGAGTCC<br>CAATGTGCTCCATTCTCACG	262	88.3
KCNJ11	Subfamily J, member 11 (BIR; HHF2; PHHI; IKATP; TNDM3; KIR6.2; MGC133230)	ATCATCGTCATCCTGGAAGG<br>GGTGTTGCCAAACTTGGAGT	162	85.8
KCNJ12	Subfamily J, member 12 (IRK2, KCNJN1, Kir2.2, Kir2.2v, hIRK, hkir2.2x, kcnj12x)	TCGATGTGGGCTTCGACAA<br>GTAGAGGGCACCTCATAGG	310	87.4
KCNJ13	Subfamily J, member 13 (KIR1.4, KIR7.1, MGC33328)	TTGGGGAATCCTAATGGACA<br>CAGGGAGAAGGAGAATGCAG	211	81.6
KCNJ14	Subfamily J, member 14 (IRK4; KIR2.4; MGC46111)	CTGGTCTTCAGCGAGAACG<br>GGGACACGAGGAAGATACGA	220	89.6
KCNJ15	subfamily J, member 15 (KIR1.3, KIR4.2, MGC13584)	CCTGCCTTGAAGAAGACACC<br>AGCGAACAGGGTGAGTTTGT	274	85.6
KCNJ16	subfamily J, member 16 (BIR9; KIR5.1; MGC33717)	TTGAGCTGGGAAATCCTTTG<br>TTGCGTCCGCATTGATAATA	187	79.5
**Large conductance calcium-activated channel**				
KCNMA1	subfamily M, alpha member 1 (MaxiK, SAKCA, SLO, SLO-ALPHA)	GGAATGGGAGACGCTTCATA<br>CCTGCAGCGAAGTATCATCA	155	80.2
KCNMB1	subfamily M, beta member 1(K(VCA) beta, SLO-BETA, hslo-beta)	CTTTGCCTGGGTGTAACCAT<br>CTGGTCCCTGATGTTGGTCT	141	82.9
KCNMB2	subfamily M, beta member 2 (MaxiK channel beta 2 subunit)	AATCACACTCCTGCGCTCATACAT<br>TTCTGTGTGGTAGAGGAGGAGC	202	83.0
KCNMB3	subfamily M, beta member 3 (Maxi K beta 3 subunit)	TGCCTCAGGGAAGAAGAGAG<br>AGTGCACACCACAGGTGAAG	269	84.0
KCNMB4	subfamily M, beta member 4 (calcium activated potassium channel beta 4 subunit)	GATTGGTTCCCAGCCATTTA<br>TACAAGCCTCCTTCCCCTTT	232	85.2
**Intermediate/small conductance calcium-activated channel**				
KCNN1	subfamily N, member 1 (SK1; hSK1; SKCA1; KCa2.1)	AAGCTGAACGACCAGGCTAA<br>GTGCAGCTCCGATACAAGGT	81	81.7
KCNN2	subfamily N, member 2 (SK2; hSK2; SKCA2; KCa2.2)	AGCTGTAGTGGCAAGGAAGC<br>GCCAAGTCCACCAAAGTGTT	279	78.5
KCNN3	subfamily N, member 3 (SK3; hSK3; SKCA3; KCa2.3)	TCCTCCAAGCTATCCACCAG<br>TCCGGTCATTGAGTTCTGTG	135	80.9
KCNN4	subfamily N, member 4 (IK1, IKCA1, KCA4, KCa3.1, SK4, hIKCa1, hKCa4, hSK4)	CATCACATTCCTGACCATCG<br>ACGTGCTTCTCTGCCTTGTT	159	88.0

Molecular cloning and lentiviral miRNA construction:

miR RNAi cloning

Creation of pcDNA6.2-GW_mCherry-miR neg

Emerald GFP (emGFP: Invitrogen) was replaced in pcDNA6.2-GW_EmGFP-miR negative control vector (miR neg sequence 5'- gtctccacgcgcagtacattt-3') with mCherry (Clontech) by excising with restriction endonuclease *Dra*I and inserting PCR amplified *Dra*I digested mCherry, (Table 2: PCR primers; forward *Dra*I mCherry, reverse *Dra*I mCherry) to create pcDNA6.2-GW_mCherry-miR neg (Figure M1).

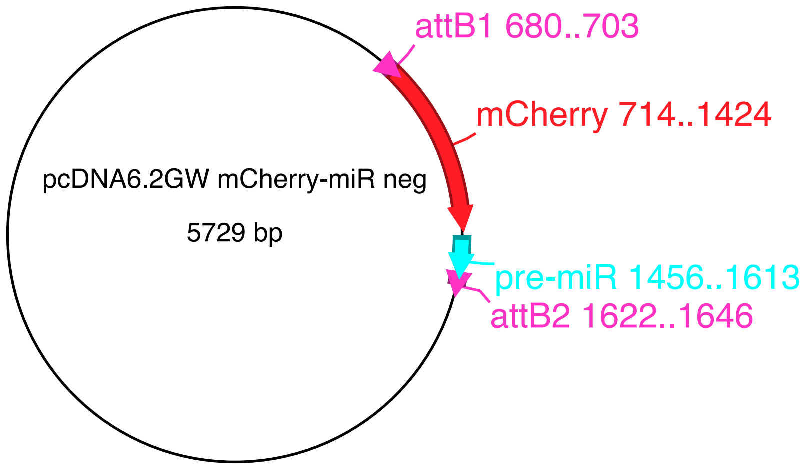

Figure M1. Plasmid map of negative control pcDNA6.2-GW_mCherry-miR neg. A GATEWAY (Invitrogen) enabled cassette flanked by recombination sites attB1 and attB2 consisted of mCherry (Clontech) and a pre-miR containing a sequence 5'-gtctccacgcgcagtacattt-3' with no similarity to the human or mouse transcriptome.

Design of pLenti6.2-GW-mCherry-KCNJ13 miR RNAi

We used the Invitrogen Block-iT RNAi designer web programme to identify DNA sequences within KCNJ13 (NM_002242) that could be incorporated into the pre-miR then aligned human (NM_002242) and mouse (NM_001110227.1) DNA sequences using Lasergene (DNAStar Inc.) and chose sequences that were also conserved between species as putative sequences to silence target genes. The miR RNAi were numbered according to their distance from the start codon (ATG) of KCNJ13; 404 5'- tcgccttacttgccatacaaa-3', h543G 5'-ggatggcaaacctaatcttat-3', m543A 5'- agatggcaaacctaatcttat-3' and 1028 5'-tcaatggacaaagcattgaca-3'. DNA sequence 543 was species specific, due to a one base-pair mismatch, necessitating construction of human (h543G) and mouse (m543A) specific sequences. These were incorporated into oligonucleotides encoding the full pre-miR expression cassette with *BamH*I and *Xho*I compatible overhangs (Table M2), annealed and inserted into *BamH*I and *Xho*I digested pcDNA6.2-GW_mCherry-miR neg replacing miR neg

to create pcDNA6.2-GW_mCherry-miR 404, -h543G, -m543A and -1028. Inserts were verified by DNA sequencing before recombining into pLenti6.2 (Figure M2).

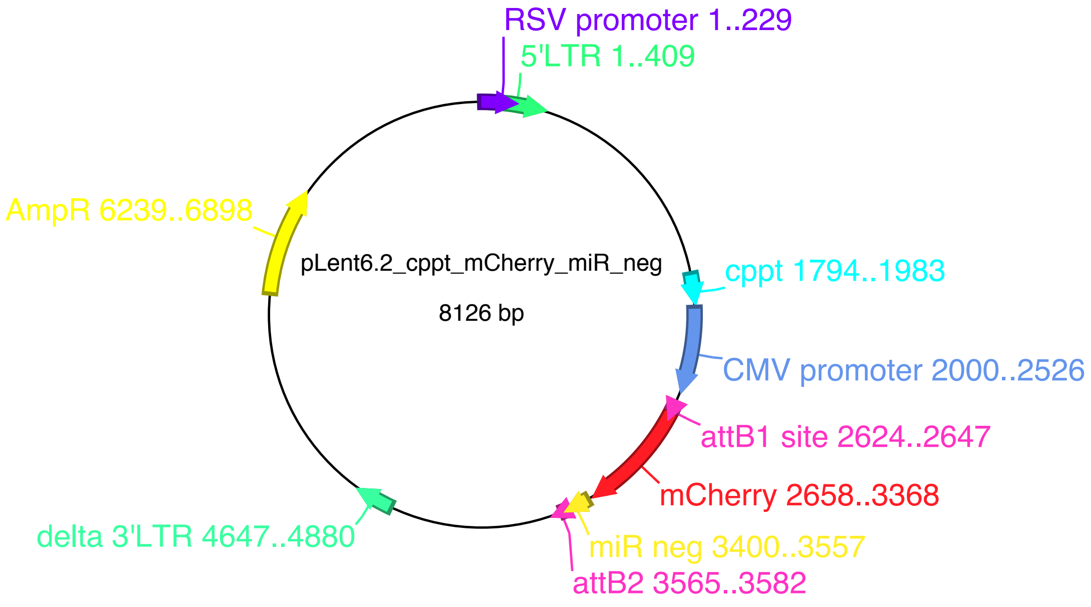

Figure M2. Plasmid map of pLenti6.2-cppt-mcherry-miR-neg. RSV, rous sarcoma virus promoter; 5'LTR, 5' long terminal repeat; cppt, central polypurine track; CMV, cytomegalovirus promoter; attB1 and attB2, recombination sites; delta 3'LTR, modified 3' long terminal repeat; AmpR, ampicillin resistance.

Cloning KCNJ13

pDONR221-KCNJ13 (Invitrogen) was converted using conventional PCR and laboratory techniques to match the protein coding region of human KCNJ13 (NM_002242). Three nucleotide mismatches were contained within a 688 bp fragment bounded by *Acc65*I and *Bgl*II restriction enzyme digestion sites. This fragment was amplified from the Invitrogen

clone using forward *Acc65*I and reverse *Bgl*II PCR primers containing bases that matched the NM_002242 sequence. The PCR product was gel purified then further amplified with GATEWAY enabled PCR primers attB1_*Acc65*I and attB5R_*Bgl*II gel purified and recombined into pDONR221-P1-P5r to generate pDONR221-B1-*Acc65*I_KCNJ13_*Bgl*II-B5r and verified by DNA sequencing. The KCNJ13 (Invitrogen) clone was digested with *Acc65*I and *Bgl*II and the 688 bp insert replaced with the corrected PCR amplified fragment, which was obtained by digestion and gel purification of *Acc65*I_KCNJ13_*Bgl*II to create pDONR221-B1-wtKCNJ13-B5r (wt; wild-type). Two IRES-mCherry fragments were amplified by PCR from plasmid pLVX-IRES-mCherry (Clontech) using two primer pairs: attB1-IRES-mCherry and attB2-mcherry and attB5-IRES-mCherry and attB2-mcherry (Table M2) and recombined into pDONR221 P1-P2 and pDONR221 P5-P2 respectively to create pDONR221 B1-IRES-mCherry-B2 and pDONR221 B5-IRES-mCherry-B2. Clones were recombined with plenti6.2-cppt-DEST-opre to create negative control; pLenti6.2-cppt-IRES-mCherry-opre (Figure M3) and pDONR221-B1-wt KCNJ13-B5r to create pLenti6.2-cppt-wtKCNJ13-IRES-mCherry-opre (Figure M4).

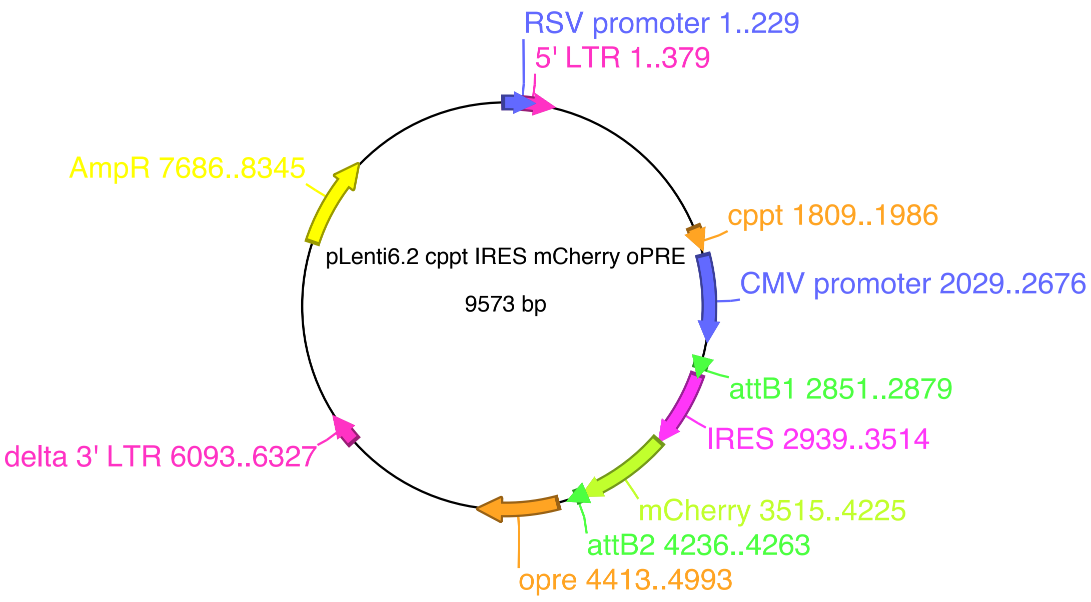

Figure M3. Plasmid map of pLenti6.2-cppt-IRES-mcherry-opre. RSV, rous sarcoma virus promoter; 5'LTR, 5' long terminal repeat; cppt, central polypurine track; CMV, cytomegalovirus promoter; attB1 and attB2, recombination sites; IRES, internal ribosome entry site; opre, optimized post-translational response element; delta 3'LTR, modified 3' long terminal repeat; AmpR, ampicillin resistance.

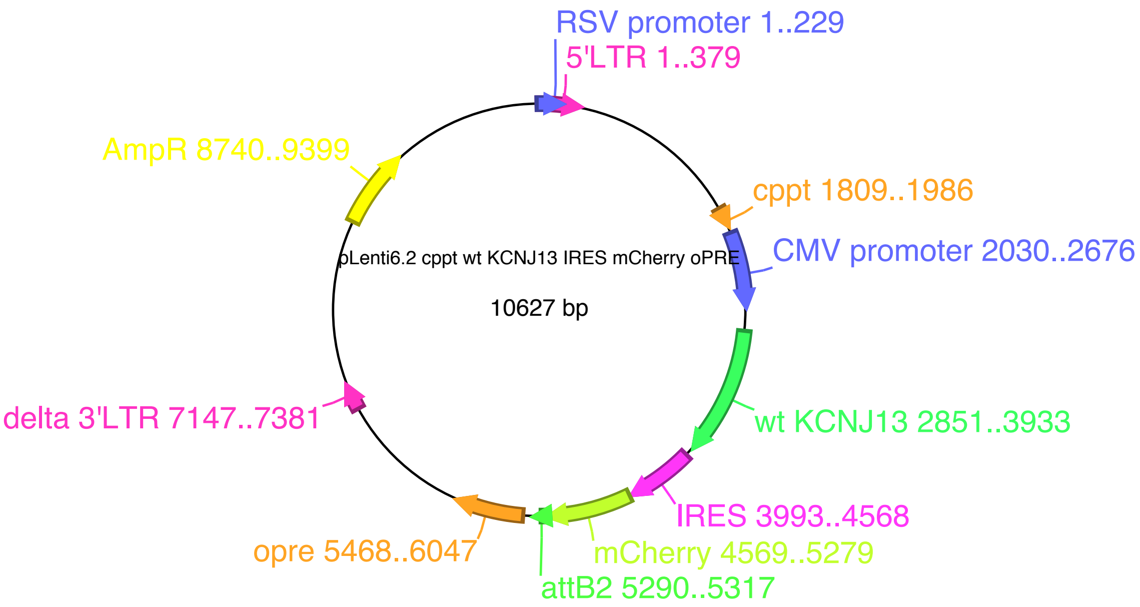

Figure M4. Plasmid map of pLenti6.2-cppt-wt KCNJ13-IRES-mcherry-opre. RSV, rous sarcoma virus promoter; 5'LTR, 5' long terminal repeat; cppt, central polypurine track; CMV, cytomegalovirus promoter; attB1 and attB2, recombination sites; wt, wild-type; KCNJ13, coding region of human potassium inwardly-rectifying channel, subfamily J, member 13; IRES, internal ribosome entry site; opre, optimized post-translational response element; delta 3'LTR, modified 3' long terminal repeat; AmpR, ampicillin resistance.

Generation of Lentivirus

Lentiviral particles Lv-mCherry-miR-neg; Lv-mCherry-KCNJ13-404; Lv-mCherry-hKCNJ13-543G; Lv-mCherry-mKcnj13-543A, Lv-mCherry-KCNJ13-1028 and Lv-KCNJ13-IRES-mCherry were generated for *in vitro* use as described in Evans et al. *Faseb J* 23, 2165-75, 2009. We generated lentivirus for *in vivo* experiments as follows: 1 x 10^7 HEK293T cells were seeded into 150 mm plates coated with a dried Matrigel basement membrane (Becton Dickinson), previously diluted 1:30 in PBS, and transfected as described (Sena-Esteves et al. *J. Virol Meth.* 122, 131-139, 2004) with a ratio of 28.35 mg pLenti6.2 shuttle vector: 20.25 mg psPAX2 (Addgene): 8.1 mg pVSV-G and left 16 h before washing 2 x with opti-MEM (Invitrogen) and cultured in 18 ml opti-MEM for a further 48 h with a change of media at 24 h. The harvests were centrifuged at 500 *g* filtered through a 0.45 mm filter (Millipore), pooled and concentrated using a centrifugal filter with a 100,000 molecular weight cut off (Millipore). Volumes were scaled up to generate a final titre of 1 x 10^8 transduction units/ml (TU/ml). Lentiviruses were aliquoted and stored at -80°C before tittering on HT1080 cells. Briefly, 2 x 10^5 HT1080 cells were plated per well of a 6 well plate and serially diluted virus added with 6 mg/ml polybrene (Sigma) and left for a minimum of 6 h before washing off and culturing cells for a further 72 h. Fluorescent cells were counted to calculate a titre, which was confirmed by a multiplicity of infection (MOI) test by adding 1, 2 and 5 viruses per HT1080 cell plated and imaged after 72 h ensuring 1:1 cellular transduction. Only titres in excess of 1 x 10^8 were used in subsequent experiments.

Table M2. Synthetic Oligonucleotides used for PCR and miR RNAi experiments

PCR	Name	DNA Sequence (5'-3')

	forward *Dra*I mCherry*	GGGGG*TTTAAA*CACCATGGTGAGCAAGGGCGAGGAG
	reverse *Dra*I mCherry	GGGG*TTTAAA*CGTTCACTTGTACAGCTCGTCCATG
	forward *Acc65*I^	GGTTAT*GGTACC*ATGTTCCCCAGTGGTGACTGTCCAAGTGCAATCGCCTTACTTGCCATACAAATGCTCCTAGGCCTCATGCTAGAGGCTTTTATCACAGGTGCTTTTGTGGCGAAGATTGCCCGGCCAAAAAATCGAGCTTTTTCAATTCGCTTTACTGACACAGCAGTAGTAG
	reverse *Bgl*II	CAG*AGATCT*GAAAATTGTCAATGCTTTGTCCATTGATGTGGATATCCAGGTCAGTCCTGTTTGGGCTTTTAGAAACCAGAGGAGTTGGAAATTCAGGGACAGTCTTGTCAAAATTCTCCATCTTGATTTGATATTCACCTTTGGAACCTCGGGTCAACAGAGATGCAAAACAGTGATGTAACATGATTTCAGACGGTAGG
	attB1_*Acc65*I	GGGGACAAGTTTGTACAAAAAAGCAGGCTGGTTAT*GGTACC*ATGTTCCCCAG
	attB5R_*Bgl*II	GGGGACAACTTTTGTATACAAAGTTGTCAG*AGATCT*GAAAATTGTCAATGC
	attB1-IRES-mCherry	GGGGACAAGTTTGTACAAAAAAGCAGGCTACTAGAGGATCTATTTCCGGTG
	attB2-mcherry	GGGGACCACTTTGTACAAGAAAGCTGGGTCAGACGCGTTCACTTGTACAG
	attB5-IRES-mCherry	GGGGACAACTTTGTATACAAAAGTTGACTAGAGGATCTATTTCCGGTG
miR RNAi	KCNJ13 404 top	*GATCC*TGGAGGCTTGCTGAAGGCTGTATGCTGTTTGTATGGCAAGTAAGGCGAGTTTTGGCCACTGACTGACTCGCCTTATGCCATACAAACAGGACACAAGGCCTGTTACTAGCACTCACATGGAAC

		AAATGGCCCAGATCTGGCCGCA*C*
	KCNJ13 404 bottom	*TCGAG*TGCGGCCAGATCTGGGCCATTTGTTCCATGTGAGTGCTA GTAACAGGCCTTGTGTCCTGTTTGTATGGCATAAGGCGAGTCA GTCAGTGGCCAAAACTCGCCTTACTTGCCATACAAACAGCATA CAGCCTTCAGCAAGCCTCCA*G*
	KCNJ13 h543G top	*GATCC*TGGAGGCTTGCTGAAGGCTGTATGCTGATAAGATTAGG TTTGCCATCCGTTTTGGCCACTGACTGACGGATGGCACCTAATC TTATCAGGACACAAGGCCTGTTACTAGCACTCACATGGAACAA ATGGCCCAGATCTGGCCGCA*C*
	KCNJ13 h543G bottom	*TCGAG*TGCGGCCAGATCTGGGCCATTTGTTCCATGTGAGTGCTA GTAACAGGCCTTGTGTCCTGATAAGATTAGGTGCCATCCGTCA GTCAGTGGCCAAAACGGATGGCAAACCTAATCTTATCAGCATA CAGCCTTCAGCAAGCCTCCA*G*
	KCNJ13 m543A top	*GATCC*TGGAGGCTTGCTGAAGGCTGTATGCTGATAAGATTAGG TTTGCCATCTGTTTTGGCCACTGACTGACAGATGGCACCTAATC TTATCAGGACACAAGGCCTGTTACTAGCACTCACATGGAACAA ATGGCCCAGATCTGGCCGCA*C*
	KCNJ13 m543A bottom	*TCGAG*TGCGGCCAGATCTGGGCCATTTGTTCCATGTGAGTGCTA GTAACAGGCCTTGTGTCCTGATAAGATTAGGTGCCATCTGTCA GTCAGTGGCCAAAACAGATGGCAAACCTAATCTTATCAGCATA CAGCCTTCAGCAAGCCTCCA*G*
	KCNJ13 1028 top	*GATCC*TGGAGGCTTGCTGAAGGCTGTATGCTGTGTCAATGCTTT GTCCATTGAGTTTTGGCCACTGACTGACTCAATGGAAAGCATT GACACAGGACACAAGGCCTGTTACTAGCACTCACATGGAACA AATGGCCCAGATCTGGCCGCA*C*
	KCNJ13 1028 bottom	*TCGAG*TGCGGCCAGATCTGGGCCATTTGTTCCATGTGAGTGCTA GTAACAGGCCTTGTGTCCTGTGTCAATGCTTTCCATTGAGTCAG

		TCAGTGGCCAAAACTCAATGGACAAAGCATTGACACAGCATA CAGCCTTCAGCAAGCCTCCA*G*

*Dra*I, *Acc65*I, *Bgl*II, *BamH*I and *Xho*I restriction enzyme sites are italicized (*) and DNA nucleotides (^) altered to match NM_002242 are underlined.

Compound screening

Cell line generation;

hKir7.1 (NM_002242.4*)* expressing Chinese hamster ovary (CHO) cells were generated using the Flp-In system (Invitrogen)[1]. Flp-in host CHO cells were transfected with pOG44 (3.6µg) and hKir7.1-pcDNA5-FRT (0.4µg) plasmids using Lipofectamine 2000 according to the manufacturer's guidelines. After 48 hours cells were harvested and cultured in media (see cell culture) containing Hygromycin-B (400µg/ml). After approximately 14 days under selection resultant cell colonies were expanded and cells were tested for hKir7.1 functional responses using the automated electrophysiology assay.

Cell culture;

Cells were routinely cultured at 37˚C in 5% $C0_2$ in Hams F12 Nutrient Mix Glutamax (Invitrogen), supplemented with 10% FBS (Invitrogen), 1X Penicillin-Streptomyocin (Invitrogen) and 200 ug/ml Hygromyocin-B (Invitrogen). Cells were not permitted to exceed 85% confluence and were passaged every 2-3 days following dissociation with 0.25% trypsin-EDTA solution (Invitrogen). Prior to assay, cells were transferred to 30˚C in 5% CO_2 and incubated for 90 - 120h.

Kir7.1 Automated Electrophysiology Assay;

Standard methodologies of the IonWorks®(Molecular Devices™) automated patch clamp electrophysiology system were followed as described[2]. Using the population patch clamp

(PPC) configuration[3], an ensemble average of the current from up to 64 cells per well was recorded in a 384-well microtitre plate.

External (4mM NaCl, 50 mM RbCl, 90mM KCl, 10mM HEPES, 1mM MgCl2, 1.8mM CaCl2 , pH7.3) and internal (90mM K-gluconate, 40mM KCl, 10mM NaCl, 3.2mM EGTA, 5mM HEPES, 3.2mM MgCl2, pH 7.3) recording solutions were prepared (Sigma-Aldrich) and electrical access achieved using amphotericin (200mg/ml) in the internal solution.

Cells were initially held for a period of 1s at -130mV before a ramp to +60mV was applied over a 1s period. This was followed by a 200ms step from -30 to 0mV. An identical voltage protocol was applied before and after (5-7min) application of test compounds.

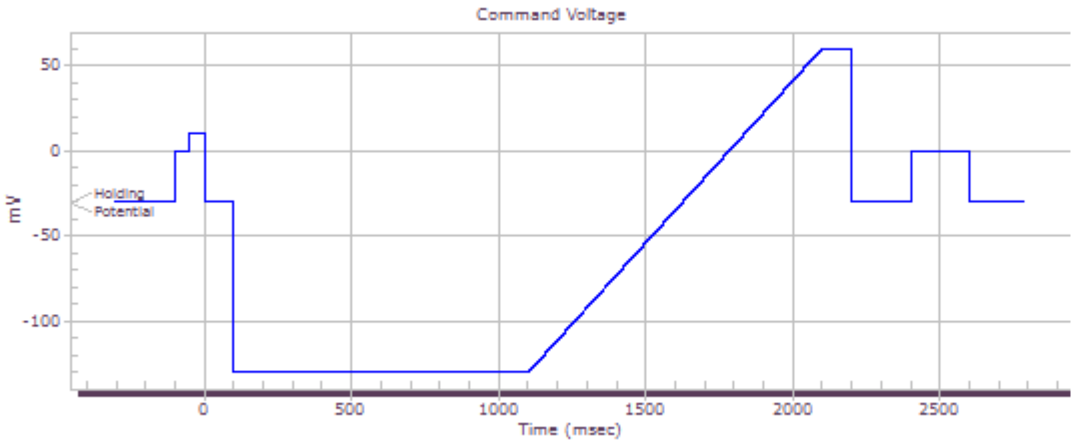

Data analysis;

Peak current amplitude was determined by subtracting the baseline current at 0 mV (2500 to 2590 ms) from the current evoked at -130 mV (800 to 1090 ms). Functional responses were expressed as a ratio of current amplitudes in the presence and absence of test compound and subsequently normalised (% inhibition) to control well responses within a plate (DMSO and 3.3mM Ba^{2+}).

$$Response = \left(\frac{Presence}{Absence}\right) \times 100$$

$$\%\ Inhibition = \left(\frac{DMSO\ Control\ Response - Test\ Compound\ Response}{DMSO\ Control\ Response - Barium\ Control\ Response}\right) \times 100$$

Non-linear regression of concentration-effect curves was performed using the 4 parameter logistic model to yield estimates of pIC50 and slope value (XLfit Version 5.1.0.0).

References;

O'Gorman *et al*, (1991). Science, **251**(4999); 1351-1355

Schroeder *et al*, (2003). J. Biomol. Screen., **8**; 50-64

Finkel *et al*, (2006). J. Biomol. Screen., **11**; 488-96
