# Supplementary material for: The inwardly rectifying K+ channel KIR7.1 controls uterine excitability throughout pregnancy
Source: EMBO Mol Med. 2014 Jul 23;6(9):1161–74. doi: 10.15252/emmm.201403944 (PMC4197863; doi:10.15252/emmm.201403944)
Supplement: Supplementary file 9 — Supplementary Data [file emmm0006-1161-SD9.pdf]

Supplementary Mathematical Methods

We constructed a mathematical model based on individual currents carried by the electrogenic transmembrane proteins in myometrium smooth muscle cell (MSMC). The aim is to analyse in detail the contributions made by these various proteins to the myometrial action potential (MAP). Our approach consists of four basic steps: (i) characterise the potential repertoire of electrogenic proteins by means of expression studies; (ii) model and parametrise each ion channel or pump independently on the basis of independent data obtained from heterologous expression studies in the literature; (iii) characterise all combinations of expression levels of these entities that are consistent with the calcium and membrane potential waveform that we observe in human MSMCs; (vi) select an expression vector based on a parsimony criterion and run a simulation based on these values.

The first step is the construction of the complete repertoire of the electrogenic proteins that are potentially expressed in MSMC based on mRNA expression data [1] (Figure D1). The molecular biology yields a list of ion channel subunits that are expressed in MSMC. Since ion channels exist as a various combinations of those subunits, this list generates a sizeable repertoire that may be present in the plasma membrane of the MSMC.

The second step is to formulate a mathematical model for every oligomeric channel complex that has been previously attested in the literature and, moreover that is consistent with the subunits in the mRNA expression list [1]. For each conductance, a mathematical model was taken from the literature where available otherwise, a model was formulated on the basis of the available data. Accordingly, the biophysical and kinetic parameters are in some cases adopted directly in the literature and in other cases obtained by means of least-squares fitting to the experimental data on heterologous expression systems as provided by the literature. The gating kinetics of each individual conductance species is represented using either a Hodgkin-Huxley model or a Markov model. The parameters and equations used to describe the kinetics of the individual conductances are listed below.

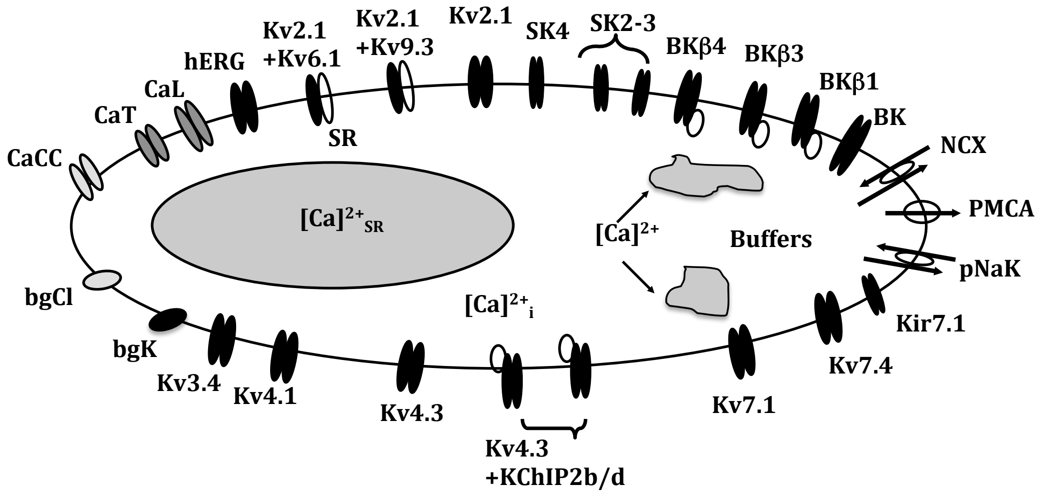

Figure D1: Myomerium cell model

Estimation of ion channel densities

With the dynamics of the individual conducting entities established, the dynamics of the membrane potential can be formulated via the standard current summation approach. The general equations and parameters describing the myometriocyte membrane potential V_m are as follows:

$$C\frac{d}{dt}V = I(t) + \sum_{i=1}^{n} \varkappa_i \psi_i(t) \tag{1}$$

$$\psi_i = g_i(\boldsymbol{x})\,(V_i - V) \tag{2}$$

$$\frac{d}{dt}\boldsymbol{x} = \boldsymbol{f}(\boldsymbol{x}, V(t), \boldsymbol{u}(t)) \tag{3}$$

The gating dynamics can be numerically integrated using the experimentally observed time series for the membrane potential together with the calcium time series. Integration of equation (1) gives an expression for the membrane potential at time t in terms of these cumulative charges:

$$V_m(t) = V_m(0) + V_0(t) + \sum_{i=1}^{n} \frac{\varkappa_i}{C} q_i(t) \tag{4}$$

where the voltage term due to injected current is given by

$$V_0(t) = \frac{1}{C}\int_0^t I(\tau)d\tau\ . \tag{5}$$

The aim is to choose the scaled densities κ_1, κ_2, ..., κ_n such that the discrepancy between the membrane potential calculated according to equation (4) is minimised. Suppose that the membrane potential has been observed at times t_1, t_2, ..., t_m, such that $m > n$. Let $\boldsymbol{W}$ be an $m \times n$ matrix whose h, ith element is defined by $W_{hi} = q_i(t_h)$. Also, let $\boldsymbol{Y}$ be an m-vector whose hth element is $V_m(t_h) - V_m(0) - V_0(t)$, and let $\boldsymbol{\kappa}$ be an n-vector collecting the scaled densities κ_i. Then the channel densities (collected in $\boldsymbol{\kappa}$) are constrained by the matrix equation $\boldsymbol{Y} = \boldsymbol{W}\cdot\boldsymbol{\kappa}$. This equation can be solved in the least-squares sense, but uniqueness is not ensured when the rank r of $\boldsymbol{W}$ is smaller than n.

To proceed, consider the singular-value decomposition $\boldsymbol{W} = \boldsymbol{U} \cdot \Sigma \cdot \boldsymbol{V}^T$. Let $\boldsymbol{V}_r$ denote the matrix containing the first r columns of the $n \times n$ matrix $\boldsymbol{V}$, let $\boldsymbol{D}$ denote an $r \times r$ diagonal matrix containing the non-zero singular values, and let $\boldsymbol{U}_r$ denote the matrix containing the first r columns of the $m \times m$ matrix $\boldsymbol{U}$. The pseudo-inverse of $\boldsymbol{W}$ is given by $\boldsymbol{V}_r \cdot \boldsymbol{D}^{-1} \cdot \boldsymbol{U}_r^T$ and the smallest-length least-squares solution is given by:

$$\boldsymbol{\kappa}^+ = \boldsymbol{V}_r \cdot \boldsymbol{D}^{-1} \cdot \boldsymbol{U}_r^T \cdot \boldsymbol{Y}\ . \tag{6}$$

Other least-squares solutions can be characterised as follows: let $\boldsymbol{v}_1^0$, $\boldsymbol{v}_2^0$, $\ldots \boldsymbol{v}_{n-r}^0$ denote the final $n - r$ columns of $\boldsymbol{V}$, which constitute an orthonormal basis for the Null space of $\boldsymbol{W}$. The general least-squares estimate for the scaled densities is then written as follows:

$$\widehat{\boldsymbol{\kappa}} = \boldsymbol{\kappa}^+ + \sum_{j=1}^{n-r} \gamma_j \boldsymbol{v}_j^0 \tag{7}$$

where the coefficients γ_1, γ_2, ..., γ_{n-r} remain to be determined.

Determining the coefficients

To single out one vector in the set of channel density vectors, further constraints must be imposed. A natural requirement is that the elements of $\widehat{\boldsymbol{\kappa}}$ be non-negative. The solutions generated by equation (7) do not generally satisfy the non-negativity. Accordingly, a vector $\boldsymbol{\kappa}_p$ was found using constrained least-squares fitting that satisfies this requirement. This vector was used instead of $\boldsymbol{\kappa}^+$ in equation (7) to generate the most parsimonious solution (the solution that satisfy the parsimony relative to total channel density) by means of linear programming. The resulting $\widehat{\boldsymbol{\kappa}}$ represents the expression profile that effects the observed behaviour with the least amount of molecular building blocks expended. The above technique will be explained in detail in a forthcoming publication.

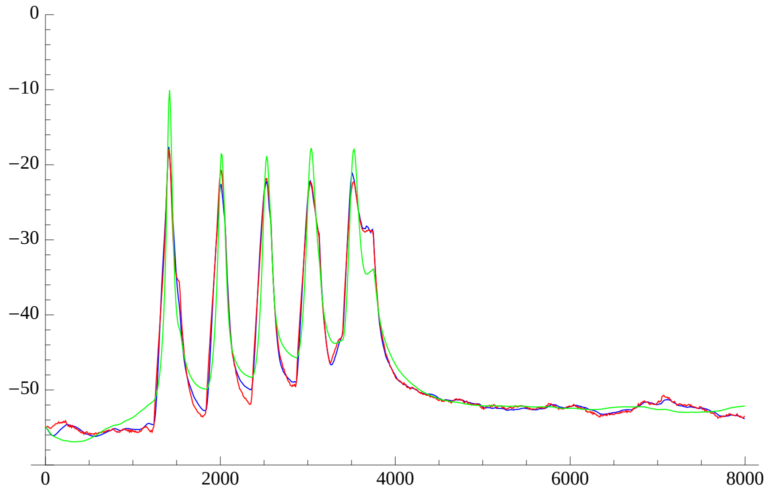

Figure D2: Red: empirical time series of the membrane potential. Blue: fit to the membrane using any set of channel density vectors $\widehat{\boldsymbol{\kappa}}$ satisfying equation (7). Green: fit to the membrane potential using any set of channel density vectors $\widehat{\boldsymbol{\kappa}}$ satisfying equation (7) with $\boldsymbol{\kappa}_p$ instead of $\boldsymbol{\kappa}^+$.

Reversal potentials:

$$\begin{aligned} E_{\mathrm{K}} &= \frac{RT}{F}\ln\frac{[\mathrm{K}^+]_o}{[\mathrm{K}^+]_i} \\ E_{\mathrm{Ca}} &= \frac{RT}{2F}\ln\frac{[\mathrm{Ca}^{2+}]_o}{[\mathrm{Ca}^{2+}]_i} \\ E_{\mathrm{Cl}} &= \frac{RT}{F}\ln\frac{[\mathrm{Cl}^-]_i}{[\mathrm{Cl}^-]_o} \end{aligned}$$

Delayed rectifier (Voltage-gated) potassium channel Kv2.1 - Based on [2]

Channel current (pA/pF)

$$I_{Kv2.1} = \kappa_{Kv2.1} G_{Kv2.1} O^{[Kv2.1]}(V - E_{\mathrm{K}})$$

Table D1: General Model Parameters

Parameters	Definition	Value
V	Membrane potential	mV
R	Gas constant	8.3143×10^3 mV C mol^{-1}K^{-1}
T	Temperature	310 K
F	Faraday constant	96,4867
C_m	Cell capacitance per unit surface area	2 μ F cm$^-2$
$[K^+]_o$	Extracellular K^+ concentration	5.4 mM
$[Na^+]_o$	Extracellular Na^+ concentration	140 mM
$[Ca^{2+}]_o$	Extracellular Ca^{2+}	2 mM

Voltage-dependent rates (s^{-1})

$$
\begin{aligned}
k_v^{[Kv2.1]} &= 12\, e^{0.77 \ \frac{VF}{RT}} \\
k_{-v}^{[Kv2.1]} &= 42\, e^{-0.54 \ \frac{VF}{RT}} \\
k_o^{[Kv2.1]} &= 80 \\
k_{-o}^{[Kv2.1]} &= 31\, e^{-0.5 \ \frac{VF}{RT}} \\
k_1^{[Kv2.1]} &= 1.2 \\
k_{-1}^{[Kv2.1]} &= 0.005
\end{aligned}
$$

$$\frac{dC_0^{[\mathrm{Kv2.1}]}}{dt} = 1-(O^{[\mathrm{Kv2.1}]}+\sum_{i=1}^{4}C_i^{[\mathrm{Kv2.1}]}+\sum_{i=0}^{5}I_i^{[\mathrm{Kv2.1}]})$$

$$\frac{dC_1^{[\mathrm{Kv2.1}]}}{dt} = 4k_v^{[\mathrm{Kv2.1}]}C_0^{[\mathrm{Kv2.1}]}-(k_{-v}^{[\mathrm{Kv2.1}]}+3k_v^{[\mathrm{Kv2.1}]}+k_1^{[\mathrm{Kv2.1}]}f^3)C_1^{[\mathrm{Kv2.1}]} +2k_{-v}^{[\mathrm{Kv2.1}]}C_2^{[\mathrm{Kv2.1}]}+\frac{k_{-1}^{[\mathrm{Kv2.1}]}}{f^3}I_1^{[\mathrm{Kv2.1}]}$$

$$\frac{dC_2^{[\mathrm{Kv2.1}]}}{dt} = 3k_v^{[\mathrm{Kv2.1}]}C_1^{[\mathrm{Kv2.1}]}-(2k_{-v}^{[\mathrm{Kv2.1}]}+2k_v^{[\mathrm{Kv2.1}]}+k_1^{[\mathrm{Kv2.1}]}f^2)C_2^{[\mathrm{Kv2.1}]} +3k_{-v}^{[\mathrm{Kv2.1}]}C_3^{[\mathrm{Kv2.1}]}+\frac{k_{-1}^{[\mathrm{Kv2.1}]}}{f^2}I_2^{[\mathrm{Kv2.1}]}$$

$$\frac{dC_3^{[\mathrm{Kv2.1}]}}{dt} = 2k_v^{[\mathrm{Kv2.1}]}C_2^{[\mathrm{Kv2.1}]}-(3k_{-v}^{[\mathrm{Kv2.1}]}+k_v^{[\mathrm{Kv2.1}]}+k_1^{[\mathrm{Kv2.1}]}f)C_3^{[\mathrm{Kv2.1}]} +4k_{-v}^{[\mathrm{Kv2.1}]}C_4^{[\mathrm{Kv2.1}]}+\frac{k_{-1}^{[\mathrm{Kv2.1}]}}{f}I_3^{[\mathrm{Kv2.1}]}$$

$$\frac{dC_4^{[\mathrm{Kv2.1}]}}{dt} = k_v^{[\mathrm{Kv2.1}]}C_3^{[\mathrm{Kv2.1}]}-(4k_{-v}^{[\mathrm{Kv2.1}]}+k_o^{[\mathrm{Kv2.1}]}+k_1^{[Kv21]})C_4^{[\mathrm{Kv2.1}]} +k_{-o}^{[\mathrm{Kv2.1}]}O_4^{[\mathrm{Kv2.1}]}+k_{-1}^{[Kv21]}I_4^{[Kv21]}$$

$$\frac{dI_0^{[\mathrm{Kv2.1}]}}{dt} = k_1^{[\mathrm{Kv2.1}]}f^4C_0^{[\mathrm{Kv2.1}]}-(\frac{k_{-1}^{[\mathrm{Kv2.1}]}}{f^4}+4\frac{k_v^{[Kv21]}}{f})I_0^{[\mathrm{Kv2.1}]} +k_{-v}^{[\mathrm{Kv2.1}]}fI_1^{[\mathrm{Kv2.1}]}$$

$$\frac{dI_1^{[\mathrm{Kv2.1}]}}{dt} = 4\frac{k_v^{[\mathrm{Kv2.1}]}}{f}I_0^{[\mathrm{Kv2.1}]}+k_1^{[\mathrm{Kv2.1}]}f^3C_1^{[\mathrm{Kv2.1}]}-(fk_{-v}^{[\mathrm{Kv2.1}]}+\frac{k_{-1}^{[\mathrm{Kv2.1}]}}{f^3} +3\frac{k_v^{[\mathrm{Kv2.1}]}}{f})I_1^{[\mathrm{Kv2.1}]}+2k_{-v}^{[\mathrm{Kv2.1}]}fI_2^{[Kv21]}$$

$$\frac{dI_2^{[\mathrm{Kv2.1}]}}{dt} = 3\frac{k_v^{[\mathrm{Kv2.1}]}}{f}I_1^{[\mathrm{Kv2.1}]}+k_1^{[\mathrm{Kv2.1}]}f^2C_2^{[\mathrm{Kv2.1}]}-(2fk_{-v}^{[\mathrm{Kv2.1}]}+\frac{k_{-1}^{[\mathrm{Kv2.1}]}}{f^2} +2\frac{k_v^{[\mathrm{Kv2.1}]}}{f})I_2^{[\mathrm{Kv2.1}]}+3k_{-v}^{[\mathrm{Kv2.1}]}fI_3^{[Kv21]}$$

$$\frac{dI_3^{[\mathrm{Kv2.1}]}}{dt} = 2\frac{k_v^{[\mathrm{Kv2.1}]}}{f}I_2^{[\mathrm{Kv2.1}]}+k_1^{[\mathrm{Kv2.1}]}fC_3^{[\mathrm{Kv2.1}]}-(3fk_{-v}^{[\mathrm{Kv2.1}]}+\frac{k_{-1}^{[\mathrm{Kv2.1}]}}{f} +\frac{k_v^{[\mathrm{Kv2.1}]}}{f})I_3^{[\mathrm{Kv2.1}]}+4k_{-v}^{[\mathrm{Kv2.1}]}fI_4^{[Kv21]}$$

$$\frac{dI_4^{[\mathrm{Kv2.1}]}}{dt} = \frac{k_v^{[\mathrm{Kv2.1}]}}{f}I_3^{[\mathrm{Kv2.1}]}+k_1^{[\mathrm{Kv2.1}]}C_4^{[\mathrm{Kv2.1}]}-(4fk_{-v}^{[\mathrm{Kv2.1}]}+k_{-1}^{[\mathrm{Kv2.1}]}+ k_o^{[\mathrm{Kv2.1}]}g)I_4^{[\mathrm{Kv2.1}]}+\frac{k_{-o}^{[\mathrm{Kv2.1}]}}{g}I_5^{[\mathrm{Kv2.1}]}$$

$$\frac{dI_5^{[\mathrm{Kv2.1}]}}{dt} = k_o^{[\mathrm{Kv2.1}]}gI_4^{[\mathrm{Kv2.1}]}-(\frac{(k_{-o}^{[\mathrm{Kv2.1}]}+k_{-1}^{[\mathrm{Kv2.1}]})}{g})I_5^{[\mathrm{Kv2.1}]}+k_1^{[\mathrm{Kv2.1}]}gO^{[\mathrm{Kv2.1}]}$$

$$\frac{dO^{[\mathrm{Kv2.1}]}}{dt} = k_o^{[\mathrm{Kv2.1}]}C_4^{[\mathrm{Kv2.1}]}-(k_{-o}^{[\mathrm{Kv2.1}]}+k_1^{[\mathrm{Kv2.1}]}g)O^{[\mathrm{Kv2.1}]}+\frac{k_{-1}^{[\mathrm{Kv2.1}]}}{g}I_5^{[\mathrm{Kv2.1}]}$$

Table D2: Kv2.1 Potassium Channel

Notation	Definition	Value
$G_{\text{Kv2.1}}$	Kv2.1unitary conductance	8.5 pS
$\kappa_{\text{Kv2.1}}$	Kv2.1 channel density	estimated
$k_1^{[\text{Kv2.1}]}$	Rate constant for inactivation with all voltage sensors activated	1.2 s^{-1}
$k_{-1}^{[\text{Kv2.1}]}$	Rate constant for recovery from inactivation	0.005 s^{-1}
$k_0^{[\text{Kv2.1}]}$	Rate constant for channel opening at 0 mV	80 s^{-1}
f	Allosteric factor	0.17
g	Allosteric factor	0.02
z_{k_v}	Apparent charge associated for k_v	0.77 e
$z_{k_{-v}}$	Apparent charge associated for k_{-v}	−0.54 e
$C_0^{[\text{Kv2.1}]} - C_4^{[\text{Kv2.1}]}$	Closed states	
$O^{[\text{Kv2.1}]}$	Open state	
$I_0^{[\text{Kv2.1}]} - I_5^{[\text{Kv2.1}]}$	Inactivation states	

Delayed rectifier (Voltage-gated, heteromeric) potassium channel Kv2.1/Kv9.3 - adapted from [3]

Channel current (pA/pF)

$$I_{\text{Kv9.3}} = \kappa_{\text{Kv9.3}}\, G_{\text{Kv9.3}}\, g_1\, (0.7 g_{2_{\text{fast}}} + 0.3 g_{2_{\text{slow}}})\, (V - E_{\text{K}})$$

$$\begin{aligned}
g_{1_\infty} &= \frac{1}{1 + e^{\left(\frac{3.2-V}{21.8}\right)}} \\
g_{2_\infty} &= \frac{1}{1 + e^{\left(\frac{44.9+V}{10.4}\right)}} \\
\tau_{g_1} &= \frac{1}{e^{\left(\frac{-80.27-V}{10}\right)} + e^{\left(\frac{-137.5+V}{55}\right)}} \\
\tau_{g_{2_{\text{fast}}}} &= 630 \text{ ms} \\
\tau_{g_{2_{\text{slow}}}} &= 3100 \text{ ms}
\end{aligned}$$

Table D3: Kv2.1/Kv9.3 Potassium Channel

Notation	Definition	Value
$G_{\text{Kv9.3}}$	Kv2.1/Kv9.3 conductance	14.5 pS
$\kappa_{\text{Kv9.3}}$	Kv2.1/Kv9.3 channel density	estimated
g_{1_∞}	Steady state activation variable	
g_{2_∞}	Steady state inactivation variable	
τ_{g_1}	Activation time constant in ms	
$\tau_{g_{2_{\text{fast}}}}$	Fast inactivation time constant in ms	
$\tau_{g_{2_{\text{slow}}}}$	Slow inactivation time constant in ms	
g_1	Activation gating variable	
$g_{2_{\text{fast}}}$,$g_{2_{\text{slow}}}$	Inactivation gating variables	

Delayed rectifier (Voltage-gated, heteromeric) potassium channel Kv2.1/Kv6.1 - adapted from [4], [3]

Channel current (pA/pF)

$$I_{\text{Kv6.1}} = \kappa_{\text{Kv6.1}} G_{\text{Kv6.1}} l_1 \, l_2 (V - E_{\text{K}})$$

$$\begin{aligned}
l_{1_\infty} &= \frac{1}{1 + e^{(\frac{-9.4 - V}{11.8})}} \\
l_{2_\infty} &= \frac{1}{1 + e^{(\frac{65.9 + V}{6.4})}} \\
\tau_{l_1} &= \frac{1}{e^{(\frac{-18.21 - V}{2})} + e^{(\frac{-107 + V}{30})}} \\
\tau_{l_2} &= 32000 \text{ ms}
\end{aligned}$$

Table D4: Kv2.1/Kv6.1 Potassium Channel

Notation	Definition	Value
$G_{\text{Kv6.1}}$	Kv2.1/Kv6.1 conductance	12.5 pS
$\kappa_{\text{Kv6.1}}$	Kv2.1/Kv6.1 channel density	estimated
l_{1_∞}	Steady state activation variable	
l_{2_∞}	Steady state inactivation variable	
τ_{l_1}	Activation time constant in ms	
τ_{l_2}	Inactivation time constant in ms	
l_1	Activation gating variable	
l_2	Inactivation gating variable	

A-type (Voltage-gated) potassium channel Kv3.4 - adapted from [5]

Channel current (pA/pF)

$$I_{\text{Kv3.4}} = \kappa_{\text{Kv3.4}}\, G_{\text{Kv3.4}}\, a_1\, a_2\, (V - E_{\text{K}})$$

$$\begin{aligned}
a_{1\infty} &= \frac{1}{1 + e^{\left(\frac{19.1 - V}{11.3}\right)}} \\
a_{2\infty} &= \frac{1}{1 + e^{\left(\frac{15 + V}{7.4}\right)}} \\
\tau_{a_1} &= 40 - \frac{37.7}{1 + e^{\left(\frac{-V}{27.78}\right)}} \\
\tau_{a_2} &= 12 + 165.6 e^{\left(\frac{-V}{11.2}\right)}
\end{aligned}$$

Table D5: Kv3.4 Potassium Channel

Notation	Definition	Value
$G_{\text{Kv3.4}}$	Kv3.4 conductance	14 pS
$\kappa_{\text{Kv3.4}}$	Kv3.4 channel density	estimated
a_{1_∞}	Steady state activation variable	
a_{2_∞}	Steady state inactivation variable	
τ_{a_1}	Activation time constant in	
τ_{a_2}	Fast inactivation time constant in ms	
a_1	Activation gating variable	
a_2	Inactivation gating variable	

A-type (Voltage-gated) potassium channel Kv4.1 - adapted from [6], [7]

Channel current (pA/pF)

$$I_{\text{Kv4.1}} = \kappa_{\text{Kv4.1}}\, G_{\text{Kv4.1}}\, {b_1}^4\, (0.18\, b_{2_{\text{fast}}} + 0.42\, b_{2_{\text{inter}}} + 0.4\, b_{2_{\text{slow}}})\, (V - E_{\text{K}})$$

$$\begin{aligned}
b_{1\infty} &= \frac{1}{1 + e^{\left(\frac{-49 - V}{22.3}\right)}} \\
b_{2\infty} &= \frac{1}{1 + e^{\left(\frac{69 + V}{5}\right)}} \\
\tau_{b_1} &= 1.96 + \frac{5.5}{1 + e^{\frac{V}{12.5}}} \\
\tau_{b_{2_{\text{fast}}}} &= 15.78 + 11.47\, e^{\left(\frac{-V}{15.93}\right)} \\
\tau_{b_{2_{\text{inter}}}} &= 73.62 + 18\, e^{\left(\frac{-V}{19}\right)} \\
\tau_{b_{2_{\text{slow}}}} &= 252.2 + 0.74\, V
\end{aligned}$$

Table D6: Kv4.1 Potassium Channel

Notation	Definition	Value
$G_{\text{Kv4.1}}$	Kv4.1 conductance	5 pS
$\kappa_{\text{Kv4.1}}$	Kv4.1 channel density	estimated
b_{1_∞}	Steady state activation variable	
b_{2_∞}	Steady state inactivation variable	
τ_{b_1}	Activation time constant in ms	
$\tau_{b_{2_{\text{fast}}}}$	Fast inactivation time constant in ms	
$\tau_{b_{2_{\text{inter}}}}$	Intermediate inactivation time constant in ms	
$\tau_{b_{2_{\text{slow}}}}$	Slow inactivation time constant in ms	
b_1	Activation gating variable	
$b_{2_{\text{fast}}}$,$b_{2_{\text{inter}}}$,$b_{2_{\text{slow}}}$	Inactivation gating variables	

A-type (voltage-gated) potassium channel Kv4.3 - based on [8]

Channel current (pA/pF)

$$I_{\text{Kv4.3}} = \kappa_{\text{Kv4.3}}\, G_{\text{Kv4.3}}\, O^{[\text{Kv4.3}]}\, (V - E_{\text{K}})$$

Voltage-dependent rates (s^{-1})

$$\begin{aligned}
\alpha^{[\text{Kv4.3}]} &= 12\, e^{0.77\, \frac{VF}{RT}} \\
\beta^{[\text{Kv4.3}]} &= 42\, e^{-0.54\, \frac{VF}{RT}} \\
k_{co}^{[\text{Kv4.3}]} &= 100\, e^{0.25\, \frac{VF}{RT}} \\
k_{oc}^{[\text{Kv4.3}]} &= 300\, e^{-0.05\, \frac{VF}{RT}}
\end{aligned}$$

$$
\begin{aligned}
\frac{dC_0^{[\mathrm{Kv4.3}]}}{dt} &= 1-(O^{[\mathrm{Kv4.3}]}+\sum_{i=1}^{4}C_i^{[\mathrm{Kv4.3}]}+\sum_{i=0}^{6}I_i^{[\mathrm{Kv4.3}]}) \\
\frac{dC_1^{[\mathrm{Kv4.3}]}}{dt} &= 4\alpha^{[\mathrm{Kv4.3}]}C_0^{[\mathrm{Kv4.3}]}-(\beta^{[\mathrm{Kv4.3}]}+3\alpha^{[\mathrm{Kv4.3}]}+k_{ci}^{[\mathrm{Kv4.3}]}f^3)C_1^{[\mathrm{Kv4.3}]}+2\beta^{[\mathrm{Kv4.3}]}C_2^{[\mathrm{Kv4.3}]}+\frac{k_{ic}^{[\mathrm{Kv4.3}]}}{f^3}I_1^{[\mathrm{Kv4.3}]} \\
\frac{dC_2^{[\mathrm{Kv4.3}]}}{dt} &= 3\alpha^{[\mathrm{Kv4.3}]}C_1^{[\mathrm{Kv4.3}]}-(2\beta^{[\mathrm{Kv4.3}]}+2\alpha^{[\mathrm{Kv4.3}]}+k_{ci}^{[\mathrm{Kv4.3}]}f^2)C_2^{[\mathrm{Kv4.3}]}+3\beta^{[\mathrm{Kv4.3}]}C_3^{[\mathrm{Kv4.3}]}+\frac{k_{ic}^{[\mathrm{Kv4.3}]}}{f^2}I_2^{[\mathrm{Kv4.3}]} \\
\frac{dC_3^{[\mathrm{Kv4.3}]}}{dt} &= 2\alpha^{[\mathrm{Kv4.3}]}C_2^{[\mathrm{Kv4.3}]}-(3\beta^{[\mathrm{Kv4.3}]}+\alpha^{[\mathrm{Kv4.3}]}+k_{ci}^{[\mathrm{Kv4.3}]}f)C_3^{[\mathrm{Kv4.3}]}+4\beta^{[\mathrm{Kv4.3}]}C_4^{[\mathrm{Kv4.3}]}+\frac{k_{ic}^{[\mathrm{Kv4.3}]}}{f}I_3^{[\mathrm{Kv4.3}]} \\
\frac{dC_4^{[\mathrm{Kv4.3}]}}{dt} &= \alpha^{[\mathrm{Kv4.3}]}C_3^{[\mathrm{Kv4.3}]}-(4\beta^{[\mathrm{Kv4.3}]}+k_o^{[\mathrm{Kv4.3}]}+k_{ci}^{[\mathrm{Kv4.3}]})C_4^{[\mathrm{Kv4.3}]}+k_{-o}^{[\mathrm{Kv4.3}]}O_4^{[\mathrm{Kv4.3}]}+k_{ic}^{[\mathrm{Kv4.3}]}I_4^{[\mathrm{Kv4.3}]} \\
\frac{dI_0^{[\mathrm{Kv4.3}]}}{dt} &= k_1^{[\mathrm{Kv4.3}]}f^4C_0^{[\mathrm{Kv4.3}]}-(\frac{k_{-1}^{[\mathrm{Kv4.3}]}}{f^4}+4\frac{\alpha^{[\mathrm{Kv4.3}]}}{f})I_0^{[\mathrm{Kv4.3}]}+\beta^{[\mathrm{Kv4.3}]}fI_1^{[\mathrm{Kv4.3}]} \\
\frac{dI_1^{[\mathrm{Kv4.3}]}}{dt} &= 4\frac{\alpha^{[\mathrm{Kv4.3}]}}{f}I_0^{[\mathrm{Kv4.3}]}+k_1^{[\mathrm{Kv4.3}]}f^3C_1^{[\mathrm{Kv4.3}]}-(f\beta^{[\mathrm{Kv4.3}]}+\frac{k_{ic}^{[\mathrm{Kv4.3}]}}{f^3}+3\frac{\alpha^{[\mathrm{Kv4.3}]}}{f})I_1^{[\mathrm{Kv4.3}]} \\
&\quad +2\beta^{[\mathrm{Kv4.3}]}fI_2^{[\mathrm{Kv4.3}]} \\
\frac{dI_2^{[\mathrm{Kv4.3}]}}{dt} &= 3\frac{\alpha^{[\mathrm{Kv4.3}]}}{f}I_1^{[\mathrm{Kv4.3}]}+k_1^{[\mathrm{Kv4.3}]}f^2C_2^{[\mathrm{Kv4.3}]}-(2f\beta^{[\mathrm{Kv4.3}]}+\frac{k_{ic}^{[\mathrm{Kv4.3}]}}{f^2}+2\frac{\alpha^{[\mathrm{Kv4.3}]}}{f})I_2^{[\mathrm{Kv4.3}]} \\
&\quad +3\beta^{[\mathrm{Kv4.3}]}fI_3^{[Kv43]} \\
\frac{dI_3^{[\mathrm{Kv4.3}]}}{dt} &= 2\frac{\alpha^{[\mathrm{Kv4.3}]}}{f}I_2^{[\mathrm{Kv4.3}]}+k_1^{[\mathrm{Kv4.3}]}fC_3^{[\mathrm{Kv4.3}]}-(3f\beta^{[\mathrm{Kv4.3}]}+\frac{k_{ic}^{[\mathrm{Kv4.3}]}}{f}+\frac{\alpha^{[\mathrm{Kv4.3}]}}{f})I_3^{[\mathrm{Kv4.3}]} \\
&\quad +4\beta^{[\mathrm{Kv4.3}]}fI_4^{[Kv43]} \\
\frac{dI_4^{[\mathrm{Kv4.3}]}}{dt} &= \frac{\alpha^{[\mathrm{Kv4.3}]}}{f}I_3^{[\mathrm{Kv4.3}]}+k_{ci}^{[\mathrm{Kv4.3}]}C_4^{[\mathrm{Kv4.3}]}-(4f\beta^{[\mathrm{Kv4.3}]}+k_{ic}^{[\mathrm{Kv4.3}]})I_4^{[\mathrm{Kv4.3}]} \\
\frac{dI_5^{[\mathrm{Kv4.3}]}}{dt} &= -(k_{io}^{[\mathrm{Kv4.3}]}+k_{56}^{[\mathrm{Kv4.3}]})I_5^{[\mathrm{Kv4.3}]}+k_{io}^{[\mathrm{Kv4.3}]}O^{[\mathrm{Kv4.3}]}+k_{65}^{[\mathrm{Kv4.3}]}I_6^{[\mathrm{Kv4.3}]} \\
\frac{dI_6^{[\mathrm{Kv4.3}]}}{dt} &= k_{56}^{[\mathrm{Kv4.3}]}I_5^{[\mathrm{Kv4.3}]}-k_{65}^{[\mathrm{Kv4.3}]}I_6^{[\mathrm{Kv4.3}]} \\
\frac{dO^{[\mathrm{Kv4.3}]}}{dt} &= k_{co}^{[\mathrm{Kv4.3}]}C_4^{[\mathrm{Kv4.3}]}-(k_{oc}^{[\mathrm{Kv4.3}]}+k_{oi}^{[\mathrm{Kv4.3}]})O^{[\mathrm{Kv4.3}]}+k_{io}^{[\mathrm{Kv4.3}]}I_5^{[\mathrm{Kv4.3}]}
\end{aligned}
$$

Potassium channel Kv4.3/KChIP2(b-d) - adapted from [9]

Channel current (pA/pF)

$$I_{\mathrm{Kv4.3/KChIP2}} = \kappa_{\mathrm{Kv4.3/KChIP2}}\, G_{\mathrm{Kv4.3/KChIP2}}\, {k_1}^4\, (A_{\mathrm{fast}}\, k_{2_{\mathrm{fast}}} + A_{\mathrm{slow}}\, k_{2_{\mathrm{slow}}})\, (V - E_{\mathrm{K}})$$

Table D7: Kv4.3 Potassium Channel

Notation	Definition	Value
$G_{\text{Kv4.3}}$	Kv4.3 unitary conductance	5 pS
$\kappa_{\text{Kv4.3}}$	Kv4.3 channel density	estimated
$k_{io}^{[\text{Kv4.3}]}$	Rate constant for open state inactivation	8 s^{-1}
$k_{oi}^{[\text{Kv4.3}]}$	Rate constant for open state inactivation	60 s^{-1}
$k_{ci}^{[\text{Kv4.3}]}$	Rate constant for closed state inactivation	7 s^{-1}
$k_{ic}^{[\text{Kv4.3}]}$	Rate constant for closed state inactivation	0.08 s^{-1}
$k_{56}^{[\text{Kv4.3}]}$		5 s^{-1}
$k_{65}^{[\text{Kv4.3}]}$		4 s^{-1}
$C_0^{[\text{Kv4.3}]} - C_4^{[\text{Kv4.3}]}$	Closed states	
$O^{[\text{Kv4.3}]}$	Open state	
$I_0^{[\text{Kv4.3}]} - I_6^{[\text{Kv4.3}]}$	Inactivation states	

Kv4.3/KChIP2b:

$$
\begin{aligned}
k_{1_\infty} &= \frac{1}{1 + e^{(\frac{-2.97 - V}{12.7})}} \\
k_{2_\infty} &= \frac{1}{1 + e^{(\frac{57.4 + V}{4.78})}} \\
\alpha &= 819 + \frac{-819}{1 + e^{\ (V - 14.6)/23.4}} \quad \text{s}^{-1} \\
4\beta &= \frac{185}{1 + e^{\ (V + 74)/13.2}} \quad \text{s}^{-1} \\
\tau_{k_1} &= \frac{1000}{\alpha + 4\beta} \\
\tau_{k_{2_{\text{fast}}}} &= 54.5 + 58.84\ \frac{e^{-\ (30 + V)}}{34.3} \\
\tau_{k_{2_{\text{slow}}}} &= 92 + 710.3\ \frac{e^{-\ (30 + V)}}{28.3} \\
\frac{A_{\text{fast}}}{A_{\text{fast}} + A_{\text{slow}}} &= 0.63 + \frac{0.63}{1 + e^{\ (V - 19.3)/8.5}}
\end{aligned}
$$

Kv4.3/KChIP2d:

$$
\begin{aligned}
k_{1_\infty} &= \frac{1}{1+e^{\left(\frac{-2.3-V}{12.49}\right)}} \\
k_{2_\infty} &= \frac{1}{1+e^{\left(\frac{61.1+V}{5}\right)}} \\
\alpha &= 1044.6 + \frac{-1044.6}{1+e^{\ (V-17.3)/24}} \quad \mathrm{s}^{-1} \\
4\beta &= \frac{240.9}{1+e^{\ (V+87.65)/16.3}} \quad \mathrm{s}^{-1} \\
\tau_{k_1} &= \frac{1000}{\alpha+4\beta} \\
\tau_{k_{2_\mathrm{fast}}} &= 54.6 + 40\frac{e^{-\ (30+V)}}{17.7} \\
\tau_{k_{2_\mathrm{slow}}} &= 114.3 + 30.14\ \frac{e^{-\ (V-10)}}{5.862} \\
\frac{A_\mathrm{fast}}{A_\mathrm{fast}+A_\mathrm{slow}} &= 0.8 + \frac{0.08}{1+e^{\ (V-33.982)/4.3}}
\end{aligned}
$$

Table D8: Kv4.3/KChIP2 Potassium Channel

Notation	Definition	Value
$G_{\mathrm{Kv4.3/KChIP2}}$	Kv4.3/KChIP2(b-d) conductance	5 pS
$\kappa_{\mathrm{Kv4.3/KChIP2}}$	Kv4.3/KChIP2(b-d) channel density	estimated
A_fast,A_slow	Relative magnitude of the fast and slow inactivation kinetics	
b_{2_∞}	Steady state inactivation variable	
τ_{k_1}	Activation time constant in ms	
$\tau_{k_{2_\mathrm{fast}}}$,$\tau_{k_{2_\mathrm{slow}}}$	Fast and slow inactivation time constant in ms	
k_1	Activation gating variable	
k_{2_fast},k_{2_slow}	Fast and slow inactivation gating variable	

Voltage-gated potassium channel hERG-based on [10]

Channel current (pA/pF)

$$I_{\text{hERG}} = \kappa_{\text{hERG}}\, G_{\text{hERG}}\, O^{[\text{hERG}]}\, (V - E_{\text{K}})$$

Voltage-dependent rates (ms^{-1})

$$\begin{aligned}
\alpha_1^{[\text{hERG}]} &= 0.022348\, e^{0.01176\ V} \\
\beta_1^{[\text{hERG}]} &= 0.047002\, e^{-0.0631\ V} \\
k_f^{[\text{hERG}]} &= 0.023761 \\
k_b^{[\text{hERG}]} &= 0.036778 \\
\alpha_2^{[\text{hERG}]} &= 0.013733\, e^{0.038198\ V} \\
\beta_2^{[\text{hERG}]} &= 0.0000689\, e^{-0.04178\ V} \\
\alpha_i^{[\text{hERG}]} &= 0.090821\, e^{0.023391\ V} \\
\beta_i^{[\text{hERG}]} &= 0.006497\, e^{-0.03268\ V}
\end{aligned}$$

$$\begin{aligned}
\frac{dC_1^{[\text{hERG}]}}{dt} &= -\alpha_1^{[\text{hERG}]} C_1^{[\text{hERG}]} + \beta_1^{[\text{hERG}]} C_2^{[\text{hERG}]} \\
\frac{dC_2^{[\text{hERG}]}}{dt} &= \alpha_1^{[\text{hERG}]} C_1^{[\text{hERG}]} - (\beta_1^{[\text{hERG}]} + k_f^{[\text{hERG}]}) C_2^{[\text{hERG}]} + k_b^{[\text{hERG}]} C_3^{[\text{hERG}]} \\
\frac{dC_3^{[\text{hERG}]}}{dt} &= k_f^{[\text{hERG}]} C_2^{[\text{hERG}]} - (\alpha_2^{[\text{hERG}]} + k_b^{[\text{hERG}]}) C_3^{[\text{hERG}]} + \beta_2^{[\text{hERG}]} O^{[\text{hERG}]} \\
\frac{dO^{[\text{hERG}]}}{dt} &= \alpha_2^{[\text{hERG}]} C_3^{[\text{hERG}]} - (\beta_2^{[\text{hERG}]} + \alpha_i^{[\text{hERG}]}) O^{[\text{hERG}]} + \beta_i^{[\text{hERG}]} I^{[\text{hERG}]} \\
\frac{dI^{hERG}}{dt} &= \alpha_i^{[\text{hERG}]} O^{[\text{hERG}]} - \beta_i^{[\text{hERG}]} I^{[\text{hERG}]}
\end{aligned}$$

Table D9: hERG Potassium Channel

Notation	Definition	Value
G_{hERG}	hERG unitary conductance	2 pS
κ_{hERG}	hERG channel density	estimated
$\alpha_1^{[\text{hERG}]}$, $\alpha_2^{[\text{hERG}]}$	Forward voltage-dependent transition rate	
$\beta_1^{[\text{hERG}]}$, $\beta_2^{[\text{hERG}]}$	Backward voltage-dependent transition rate	
$k_f^{[\text{hERG}]}$	Voltage-insensitive forward rate constant	0.023761 ms^{-1}
$k_b^{[\text{hERG}]}$	Voltage-insensitive backward rate constant	0.036778 ms^{-1}
$\alpha_i^{[\text{hERG}]}$	Forward voltage-dependent inactivation rate	
$\beta_i^{[\text{hERG}]}$	Backward voltage-dependent inactivation rate	
$C_0^{[\text{hERG}]}$ –$C_2^{[\text{hERG}]}$	Closed states	
$O^{[\text{hERG}]}$	Open state	
$I_0^{[\text{hERG}]}$	Inactivation state	

Voltage-dependent potassium channel Kv7.1 - based on [11]

Channel current (pA/pF)

$$I_{\mathrm{Kv7.1}} = \kappa_{\mathrm{Kv7.1}}\, G_{\mathrm{Kv7.1}}\, P_o^{[Kv7.1]}\, (V - E_{\mathrm{K}})$$

Voltage-dependent rates (s^{-1})

$$\begin{aligned}
\alpha_1^{[\mathrm{Kv7.1}]} &= 4.6\, e^{0.47\, \frac{VF}{RT}} \\
\beta_1^{[\mathrm{Kv7.1}]} &= 33\, e^{-0.35\, \frac{VF}{RT}} \\
\alpha_2^{[\mathrm{Kv7.1}]} &= 24\, e^{0.006\, \frac{VF}{RT}} \\
\beta_2^{[\mathrm{Kv7.1}]} &= 19\, e^{-0.007\, \frac{VF}{RT}} \\
\epsilon^{[\mathrm{Kv7.1}]} &= 4.6\, e^{0.8\, \frac{VF}{RT}} \\
\delta^{[\mathrm{Kv7.1}]} &= 1.4\, e^{-0.7\, \frac{VF}{RT}}
\end{aligned}$$

$$\begin{aligned}
\frac{dC_1^{[\mathrm{Kv7.1}]}}{dt} &= -\alpha_1^{[\mathrm{Kv7.1}]} C_1^{[\mathrm{Kv7.1}]} + \beta_1^{[\mathrm{Kv7.1}]} C_2^{[\mathrm{Kv7.1}]} \\
\frac{dC_2^{[\mathrm{Kv7.1}]}}{dt} &= \alpha_1^{[\mathrm{Kv7.1}]} C_1^{[\mathrm{Kv7.1}]} - (\beta_1^{Kv7.1} + \alpha_2^{[\mathrm{Kv7.1}]}) C_2^{[\mathrm{Kv7.1}]} + \beta_2^{[\mathrm{Kv7.1}]} O_1^{[\mathrm{Kv7.1}]} \\
\frac{dO_1^{[\mathrm{Kv7.1}]}}{dt} &= \alpha_2^{[\mathrm{Kv7.1}]} C_2^{[\mathrm{Kv7.1}]} - (\epsilon^{[\mathrm{Kv7.1}]} + \beta_2^{[\mathrm{Kv7.1}]}) O_1^{[\mathrm{Kv7.1}]} + \delta^{[\mathrm{Kv7.1}]} O_2^{[\mathrm{Kv7.1}]} \\
\frac{dO_2^{[\mathrm{Kv7.1}]}}{dt} &= \epsilon^{[\mathrm{Kv7.1}]} O_1^{[\mathrm{Kv7.1}]} - (\delta^{\mathrm{Kv7.1}} + \lambda^{[\mathrm{Kv7.1}]}) O_2^{[\mathrm{Kv7.1}]} + \mu^{[\mathrm{Kv7.1}]} I^{[\mathrm{Kv7.1}]} \\
\frac{dI^{[\mathrm{Kv7.1}]}}{dt} &= \lambda^{[\mathrm{Kv7.1}]} O_2^{[\mathrm{Kv7.1}]} - \mu^{[\mathrm{Kv7.1}]} I^{[\mathrm{Kv7.1}]}
\end{aligned}$$

Table D10: Kv7.1 Potassium Channel

Notation	Definition	Value
$G_{\mathrm{Kv7.1}}$	Kv7.1 unitary conductance	1.8 pS
$\kappa_{\mathrm{Kv7.1}}$	Kv7.1 channel density	estimated
$\lambda^{[\mathrm{Kv7.1}]}$	Rate constant for open state inactivation	142 s^{-1}
$\mu^{[\mathrm{Kv7.1}]}$	Rate constant for open state inactivation	52 s^{-1}
$\alpha_1^{[\mathrm{Kv7.1}]}$, $\alpha_2^{[\mathrm{Kv7.1}]}$,$\epsilon^{[\mathrm{Kv7.1}]}$	Forward voltage-dependent transition rates	
$\beta_1^{[\mathrm{Kv7.1}]}$, $\beta_2^{[\mathrm{Kv7.1}]}$,$\delta^{[\mathrm{Kv7.1}]}$	Backward voltage-dependent transition rates	
$C_1^{[\mathrm{Kv7.1}]}$, $C_2^{[\mathrm{Kv7.1}]}$	Closed states	
$O_1^{[\mathrm{Kv7.1}]}$,$O_2^{[\mathrm{Kv7.1}]}$	Open states	
$I^{[\mathrm{Kv7.1}]}$	Inactivation state	

Voltage- dependent potassium channel Kv7.4 - adapted from [12, 13]

Channel Current (pA/pF)

$$I_{\mathrm{Kv7.4}} = \kappa_{\mathrm{Kv7.4}}\, G_{\mathrm{Kv7.4}}\, d_1\, d_2\, (V - E_{\mathrm{K}})$$

$$\begin{aligned}
d_\infty &= \frac{1}{1 + e^{\left(\frac{-32-V}{17.4}\right)}} \\
\tau_{d_{\text{fast}}} &= \frac{1}{e^{9.1(-65-V)} + e^{0.03(-132.42+V)}} \\
\tau_{d_{\text{slow}}} &= 318 \text{ ms} \\
\tau_d &= 0.35\, \tau_{d_{\text{fast}}} + 0.65\, \tau_{d_{\text{slow}}} \\
\tau_{d_{\text{deac}}} &= 7.5 \text{ ms}
\end{aligned}$$

Table D11: Kv7.4 Potassium Channel

Notation	Definition	Value
$G_{\mathrm{Kv7.4}}$	Kv7.4 conductance	2.1 pS
$\kappa_{\mathrm{Kv7.4}}$	Kv7.4 channel density	estimated
d_∞	Activation and deactivation steady state variable	
$\tau_{d_{\text{fast}}}$	Fast activation time constant in ms	
$\tau_{d_{\text{slow}}}$	Slow activation time constant in ms	
τ_d	Activation time constant in ms	
$\tau_{d_{\text{deac}}}$	Deactivation time constant in ms	
d_1	Activation gating variable	
d_2	Deactivation gating variable	

Inward Rectifier Kir7.1 - adapted from [14]

Channel Current(pA/pF)

$$I_{\text{Kir7.1}} = \kappa_{\text{Kir7.1}} G_{\text{Kir7.1}} P_{\text{Kir7.1}} g_{\text{Kir7.1}} (V - E_{\text{K}}).$$

$$\begin{aligned} P_{\text{Kir7.1}_{ss}} &= 0.083\, e^{-0.018\ V} \\ g_{\text{Kir7.1}} &= 0.65\, [\text{K}^+]_o^{0.095} \\ \tau_{\text{Kir7.1}} &= 2 + 0.01\, V \end{aligned}$$

Table D12: Kir7.1 Potassium Channel

Notation	Definition	Value
$G_{\text{Kir7.1}}$	Kir7.1 conductance	0.056 pS
$\kappa_{\text{Kir7.1}}$	Kir7.1 channel density	estimated
$g_{\text{Kir7.1}}$	The slope conductance dependence on $[\text{K}^+]_o$	
$P_{\text{Kir7.1}_{ss}}$	Steady state activation	
$\tau_{\text{Kir7.1}}$	Activation time constant in ms	
$P_{\text{Kir7.1}}$	Channel open probability	

Voltage and Calcium- dependent potassium channel BK$_{\alpha}$ and BK$_{\alpha+\beta 1}$- based on [15] and [16]

Channel Current (pA/pF)

$$I_{\text{BK}} = \kappa_{\text{BK}} G_{\text{BK}} P_{\text{BK}} (V - E_{\text{K}})$$

Closed-to-open and open-to-closed rate constants (s^{-1})

$$\begin{aligned} \delta_{xi} &= \delta_{o_i} e^{(\frac{z_\delta * FV}{RT})} \\ \gamma_{xi} &= \gamma_{o_i} e^{(\frac{z_\gamma * FV}{RT})} \text{ where } i = 1 \ldots 5 \end{aligned}$$

Equilibrium constants when the channels is closed or open

$$\begin{aligned} j_c &= e^{(\frac{z_j F (V - V_{hc})}{RT})} \\ j_o &= e^{(\frac{z_j F (V - V_{ho})}{RT})} \end{aligned}$$

Fractions of closed channels occupying the state that precedes each transition

$$f_{co} = \frac{1}{1 + 4j_c + 6{j_c}^2 + 4{j_c}^3 + {j_c}^4}$$
$$f_{c1} = \frac{4j_c}{1 + 4j_c + 6{j_c}^2 + 4{j_c}^3 + {j_c}^4}$$
$$f_{c2} = \frac{6{j_c}^2}{1 + 4j_c + 6{j_c}^2 + 4{j_c}^3 + {j_c}^4}$$
$$f_{c3} = \frac{4{j_c}^3}{1 + 4j_c + 6{j_c}^2 + 4{j_c}^3 + {j_c}^4}$$
$$f_{c4} = \frac{{j_c}^4}{1 + 4j_c + 6{j_c}^2 + 4{j_c}^3 + {j_c}^4}$$

Fractions of open channels occupying the state that precedes each transition

$$f_{oo} = \frac{1}{1 + 4j_o + 6{j_o}^2 + 4{j_o}^3 + {j_o}^4}$$
$$f_{o1} = \frac{4j_o}{1 + 4j_o + 6{j_o}^2 + 4{j_o}^3 + {j_o}^4}$$
$$f_{o2} = \frac{6{j_o}^2}{1 + 4j_o + 6{j_o}^2 + 4{j_o}^3 + {j_o}^4}$$
$$f_{o3} = \frac{4{j_o}^3}{1 + 4j_o + 6{j_o}^2 + 4{j_o}^3 + {j_o}^4}$$
$$f_{o4} = \frac{{j_o}^4}{1 + 4j_o + 6{j_o}^2 + 4{j_o}^3 + {j_o}^4}$$

$$\begin{aligned}\delta &= (\delta_{x1} * f_{co}) + (\delta_{x2} * f_{c1}) + (\delta_{x3} * f_{c2}) + (\delta_{x4} * f_{c3}) + (\delta_{x5} * f_{c4}) \\ \gamma &= (\gamma_{x1} * f_{oo}) + (\gamma_{x2} * f_{o1}) + (\gamma_{x3} * f_{o2}) + (\gamma_{x4} * f_{o3}) + (\gamma_{x5} * f_{o4})\end{aligned}$$

$$\tau_{\mathrm{BK}_{\alpha/\alpha+\beta 1}} = \frac{1}{\gamma + \delta}$$

$$P_{\mathrm{BK}_{\alpha/\alpha+\beta 1_{ss}}} = \frac{1}{1 + \left(\frac{1+e^{(z_j F(V-V_{hc})/RT)}}{1+e^{(z_j F(V-V_{ho})/RT)}}\right)^4 \left(\frac{1+[\mathrm{Ca}^{2+}]_i/K_c}{1+[\mathrm{Ca}^{2+}]_i/K_o}\right)^8 \frac{e^{(-z_L FV/RT)}}{L}}$$

Table D13: BK_α and the $\mathrm{BK}_{\alpha+\beta 1}$ Potassium Channels

Notation	Definition	Value
G_{BK}	BK unitary conductance (BK_α , $\mathrm{BK}_{\alpha+\beta 1}$)	289 pS
κ_{BK}	BK channel density (BK_α , $\mathrm{BK}_{\alpha+\beta 1}$)	estimated
$P_{o\mathrm{BK}}$	BK open channel probability (BK_α , $\mathrm{BK}_{\alpha+\beta 1}$)	
L_0	(BK_α / $\mathrm{BK}_{\alpha+\beta 1}$)	$2.2*10^{-6}$ / $2.5*10^{-6}$
z_J	Gating charge (BK_α / $\mathrm{BK}_{\alpha+\beta 1}$)	0.58e / 0.57 e
z_γ	” (BK_α / $\mathrm{BK}_{\alpha+\beta_1}$)	0.1e / 0.17 e
z_δ	” $z_L - z_\gamma$	
z_L	Gating charge associated	
	with closed-to-open conformational change	
	(BK_α / $\mathrm{BK}_{\alpha+\beta_1}$)	0.41e / 0.46 e
V_{ho}	Voltage sensor’s half activation V when channel closed	
	(BK_α / $\mathrm{BK}_{\alpha+\beta_1}$)	27 / -34 mV
V_{hc}	Voltage sensor’s half activation V when channel is open	
	(BK_α / $\mathrm{BK}_{\alpha+\beta_1}$)	151 / 80 mV
D	Allosteric factor (BK_α / $\mathrm{BK}_{\alpha+\beta_1}$)	16.8 / 12.8
δ_{0_1}	Rate constant for channel opening (BK_α / $\mathrm{BK}_{\alpha+\beta_1}$)	0.016 / 0.003 s^{-1}
δ_{0_2}	”	0.114 / 0.007 s^{-1}
δ_{0_3}	”	1.98 / 0.198 s^{-1}
δ_{0_4}	”	3.76 / 1.251 s^{-1}
δ_{0_5}	”	57.12 / 4.934 s^{-1}
γ_{0_1}	Rate constant for channel closing (BK_α / $\mathrm{BK}_{\alpha+\beta_1}$)	7452.3 / 931.7 s^{-1}
γ_{0_2}	”	4121.4 / 213.2 s^{-1}
γ_{0_3}	”	5645.8 / 547.8 s^{-1}
γ_{0_4}	”	851 / 333.5 s^{-1}
γ_{0_5}	”	1025 / 126.7 s^{-1}
$P_{\mathrm{BK}_{\alpha/\alpha+\beta 1_{ss}}}$	Steady-state activation	
$P_{o\mathrm{BK}_{\alpha/\alpha+\beta 1}}$	Open-state probability	

Voltage and Calcium- dependent potassium channel BK$_{\alpha+\beta3}$- based on [17]

Channel Current (pA/pF)

$$I_{\mathrm{BK}_{\alpha+\beta3}} = \kappa_{\mathrm{BK}_{\alpha+\beta3}} G_{\mathrm{BK}_{\alpha+\beta3}} P_{o\mathrm{BK}_{\alpha+\beta3}} (V - E_{\mathrm{K}})$$

$$P_{\mathrm{BK}_{\alpha+\beta3_o}} = \frac{(O_n^{[\alpha+\beta3]} + I_n^{[\alpha+\beta3]})}{(C_n^{[\alpha+\beta3]} + O_n^{[\alpha+\beta3]} + I_n^{[\alpha+\beta3]})}$$

Voltage-dependent rates:

$$\begin{aligned} k_f &= k_{f_0} e^{(\frac{z_f VF}{RT})} \\ k_r &= k_{r_0} e^{(\frac{z_r VF}{RT})} \\ k_b &= k_{b_0} e^{(\frac{z_b VF}{RT})} \\ k_u &= k_{u_0} e^{(\frac{z_u VF}{RT})} \end{aligned}$$

$$\begin{aligned}
\frac{dC_0^{[\alpha+\beta3]}}{dt} &= k_c\, C_1^{[\alpha+\beta3]} - (4\,[\mathrm{Ca}^{2+}]_i + k_{f_0})\, C_0^{[\alpha+\beta3]} + k_{r_0}\, O_0^{[\alpha+\beta3]} \\
\frac{dI_0^{[\alpha+\beta3]}}{dt} &= k_i\, I_1^{[\alpha+\beta3]} - (4\,[\mathrm{Ca}^{2+}]_i + k_{b_0})\, I_0^{[\alpha+\beta3]} + k_{b_0}\, O_0^{[\alpha+\beta3]} \\
\frac{dO_0^{[\alpha+\beta3]}}{dt} &= k_o\, O_1^{[\alpha+\beta3]} - (4\,[\mathrm{Ca}^{2+}]_i + k_{r_0} + k_{b_0})\, O_0^{[\alpha+\beta3]} + k_{f_0}\, C_0^{[\alpha+\beta3]} + k_{u_0}\, I_0^{[\alpha+\beta3]} \\
\frac{dC_4^{[\alpha+\beta3]}}{dt} &= [\mathrm{Ca}^{2+}]_i\, C_3^{[\alpha+\beta3]} - (4\,k_c + k_{f_4}) C_4^{[\alpha+\beta3]} + k_{r_4}\, O_4^{[\alpha+\beta3]} \\
\frac{dO_4^{[\alpha+\beta3]}}{dt} &= [\mathrm{Ca}^{2+}]_i\, O_3^{[\alpha+\beta3]} - (4\,k_o + k_{r_4} + k_{b_4}) O_4^{[\alpha+\beta3]} + k_{f_4}\, C_4^{[\alpha+\beta3]} + k_{u_4}\, I_4^{[\alpha+\beta3]} \\
\frac{dI_4^{[\alpha+\beta3]}}{dt} &= [\mathrm{Ca}^{2+}]_i\, I_3^{[\alpha+\beta3]} - (4\,k_i + k_{u_4}) I_4^{[\alpha+\beta3]} + k_{b_4}\, O_4^{[\alpha+\beta3]} \\
\frac{dC_n^{[\alpha+\beta3]}}{dt} &= (5-n)[\mathrm{Ca}^{2+}]_i\, C_{n-1}^{[\alpha+\beta3]} + (n+1) k_c\, C_{n+1}^{[\alpha+\beta3]} - (n\,k_c + (5-n-1)[\mathrm{Ca}^{2+}]_i \\
&\quad - k_{f_n}) C_n^{[\alpha+\beta3]} + k_{r_n}\, O_n^{[\alpha+\beta3]} \\
\frac{dO_n^{[\alpha+\beta3]}}{dt} &= (5-n)[\mathrm{Ca}^{2+}]_i\, O_{n-1}^{[\alpha+\beta3]} + (n+1) k_o\, O_{n+1}^{[\alpha+\beta3]} - (n\,k_o + (5-n-1)[\mathrm{Ca}^{2+}]_i - k_{r_n} \\
&\quad + k_{b_n}) O_n^{[\alpha+\beta3]} + k_{f_n}\, C_n^{[\alpha+\beta3]} + k_{u_n}\, I_n^{[\alpha+\beta3]} \\
\frac{dI_n^{[\alpha+\beta3]}}{dt} &= (5-n)[\mathrm{Ca}^{2+}]_i\, I_{n-1}^{[\alpha+\beta3]} + (n+1) k_i\, I_{n+1}^{[\alpha+\beta3]} - (n\,k_i + (5-n-1)[\mathrm{Ca}^{2+}]_i \\
&\quad - k_{u_n}) I_n^{[\alpha+\beta3]} + k_{b_n}\, O_n^{[\alpha+\beta3]} \qquad \text{where } n = 1, 2, 3
\end{aligned}$$

Table D14: $\text{BK}_{\alpha+\beta3}$ Potassium Channel

Notation	Definition	Value
$G_{\text{BK}_{\alpha+\beta3}}$	$\text{BK}_{\alpha+\beta3}$ unitary conductance	289 pS
$\kappa_{\text{BK}_{\alpha+\beta3}}$	$\text{BK}_{\alpha+\beta3}$ channel density	estimated
k_{f_0}	Closed to open transition rate at 0 mV	1 s^{-1}
k_{f_1}	”	2 s^{-1}
k_{f_2}	”	6 s^{-1}
k_{f_3}	”	250 s^{-1}
k_{f_4}	”	500 s^{-1}
k_{r_0}	Open to close transition rate at 0 mV	4000 s^{-1}
k_{r_1}	”	1200 s^{-1}
k_{r_2}	”	800 s^{-1}
k_{r_3}	”	500 s^{-1}
k_{r_4}	”	100 s^{-1}
k_{b_n}	Open to inactivated transition rate at 0 mV	900 s^{-1}
k_{b_0}	”	900 s^{-1}
k_{b_4}	”	900 s^{-1}
k_{u_n}	Inactivated to open transition rate at 0 mV	750 s^{-1}
k_{u_0}	”	750 s^{-1}
k_{u_4}	”	750 s^{-1}
z_f	Gating charge	0.72e
z_r	”	0.67 e
z_b	”	0.072 e
z_u	”	0.361 e
k_c	Closed states dissociation constant	11.2
k_o	Open states dissociation constant	0.75
k_i	Inactvation states dissociation constant	0.72
$P_{o\text{BK}_{\alpha+\beta3}}$	Open probability	

Voltage and Calcium- dependent potassium channel $\text{BK}_{\alpha+\beta4}$- based on [15] and [18]

Channel Current (pA/pF)

$$I_{\text{BK}_{\alpha+\beta4}} = \kappa_{\text{BK}_{\alpha+\beta4}} G_{\text{BK}_{\alpha+\beta4}} P_{\text{BK}_{\alpha+\beta4}} (V - E_{\text{K}})$$

$$
\begin{aligned}
P_{\text{BK}_{\alpha+\beta4_{ss}}} &= \frac{1}{1+\frac{(1+J+K+JKE)^4}{L(1+KC+JKCDE)^4}} \\
L &= L_0 e^{(\frac{-z_l FV}{RT})} \\
J &= e^{(\frac{-z_j FV}{RT})} \\
K &= \frac{[\text{Ca}^{2+}]_i}{k_c} \\
D &= e^{(\frac{-z_j F(V_{ho}-V_{hc})}{RT})} \\
C &= \frac{k_c}{k_o} \\
\tau_{\text{BK}_{\alpha+\beta_4}} &= \tau_{\text{BK}_\alpha} + 30
\end{aligned}
$$

Table D15: $\text{BK}_{\alpha+\beta4}$ Potassium Channel

Notation	Definition	Value
$G_{\text{BK}_{\alpha+\beta4}}$	$\text{BK}_{\alpha+\beta4}$ unitary conductance	289 pS
$\kappa_{\text{BK}_{\alpha+\beta4}}$	$\text{BK}_{\alpha+\beta4}$ channel density	estimated
$P_{o\text{BK}_{\alpha+\beta4}}$	Open probability	
L_0	The zero voltage value of L	3.7*10^{-8}
z_L	The partial charge of L	0.3 e
J_0	The zero voltage value of J	1
z_j	The partial charge of J	0.55 e
K_c	Calcium dissociation constant	44 μM
	(closed channel and resting voltage sensors)	
C	Allosteric factor describing interaction	
	between channel opening and calcium binding	23
K_o	Calcium dissociation constant	1.9 μM
	(open channel, resting voltage sensors)	
D	Allosteric factor describing interaction	
	between channel opening and voltage sensor activation	32
V_{ho}	Voltage sensor's half activation V when channel is open	25 mV
V_{hc}	Voltage sensor's half activation V when channel is closed	187 mV
E	Allosteric factor describing interaction	
	between calcium binding and voltage sensor activation	3.7
$P_{\text{BK}_{\alpha+\beta4_{ss}}}$	Steady-state activation variable	
$P_{o\text{BK}_{\alpha+\beta4}}$	Open-state probabiliity	

Voltage-dependent calcium channel T-type- adapted from [19]

Channel Current (pA/pF)

$$I_{\text{T-type}} = \kappa_{\text{T-type}}\, G_{\text{T-type}}\, a\, c\, (V - E_{\text{Ca}})$$

$$a_{\infty} = \frac{1}{1+e^{(\frac{-28.6-V}{8.9})}}$$

$$c_{\infty} = \frac{1}{1+e^{(\frac{72.4+V}{4.8})}}$$

$$\tau_{\text{a}} = 1.7 + \frac{9.87}{1+e^{(\frac{V+39}{7.6})}}$$

$$\tau_{\text{c}} = 13.7 + \frac{5369.7}{1+e^{(\frac{V+108.5}{11.24})}}$$

Table D16: T-type Calcium Channel

Notation	Definition	Value
$G_{\text{T-type}}$	T-type conductance	8 pS
$\kappa_{\text{T-type}}$	T-type channel density	estimated
a_{∞}	Steady state activation variable	
c_{∞}	Steady state inactivation variable	
τ_a	Activation time constant in ms	
τ_c	Inactivation time constant in ms	
a	Activation gating variable	
c	Inactivation gating variable	

Voltage-dependent calcium channel L-type- ***based on [20]***

Channel Current (pA/pF)

$$I_{L\text{-}type} = \kappa_{\text{L-type}}\, G_{\text{L-type}}\, d\, f\, f_{\text{Ca}}(V - E_{\text{Ca}})$$

$$\begin{aligned}
d_\infty &= \frac{1}{(1+e^{\frac{-(V+10)}{6.24}})(1+e^{\frac{-(V+60)}{0.024}})} \\
f_\infty &= \frac{1}{(1+e^{\frac{(V+32)}{8}})} + \frac{0.6}{(1+e^{\frac{(50-V)}{20}})} \\
\tau_d &= \frac{28.57(1-e^{\frac{-(V+10)}{6.24}})}{(1+e^{\frac{-(V+10)}{6.24}})(V+10)} \\
\tau_f &= \frac{50}{1+e^{\frac{-(V+10)^2}{881}}} \\
f_{\text{Ca}} &= \frac{1}{(1+\frac{[\text{Ca}^{2+}]_i}{0.006})}
\end{aligned}$$

Table D17: L-type Calcium Channel

Notation	Definition	Value
$G_{\text{L-type}}$	L-type conductance	25 pS
$\kappa_{\text{L-type}}$	L-type channel density	estimated
d_∞	Steady state activation variable	
f_∞	Steady state inactivation variable	
f_{Ca}	Calcium-dependent inactivation variable	
τ_d	Activation time constant in ms	
τ_f	Inactivation time constant in ms	
d	Activation gating variable	
f	Inactivation gating variable	

Calcium-dependent chloride channel CaCC ANNO1- based on [21]

Channel Current (pA/pF)

$$I_{\text{CaCC}} = \kappa_{\text{CaCC}}\, G_{\text{CaCC}}\, cc\, (V - E_{Cl})$$

$$cc_\infty = \frac{1}{1 + K_2\,(\frac{K_1^2}{[\text{Ca}^{2+}]_i{}^2} + \frac{K_1}{[\text{Ca}^{2+}]_i} + 1)}$$

$$\begin{aligned} K_1 &= 214\, e^{0.13\; FV/RT} \quad \text{nM} \\ K_2 &= 0.58\, e^{-0.24\; FV/RT} \quad \text{nM} \end{aligned}$$

$$\frac{1}{\tau_{cc}} = \frac{0.38}{\frac{K_1^2}{[\text{Ca}^{2+}]_i^2} + \frac{K_1}{[\text{Ca}^{2+}]_i} + 1} + 0.38\, K_2 \quad s^{-1}$$

Table D18: CaCC Chloride Channel

Notation	Definition	Value
G_{CaCC}	CaCC unitary conductance	7.5 pS
κ_{CaCC}	CaCC channel density	estimated
cc_∞	Steady state activation variable	
τ_{cc}	Activation time constant in ms	
cc	Activation gating variable	

Small calcium-dependent potassium channel SK2- based on [22] and [23]

Channel Current (pA/pF)

$$I_{\text{SK2}} = \kappa_{\text{SK2}} G_{\text{SK2}} P_{\text{SK2}} (V - E_{\text{K}})$$

$$P_{\text{SK2}_{ss}} = P_{0,max} \frac{[\text{Ca}^{2+}]_i^{2.2}}{(0.74)^{2.2} + [\text{Ca}^{2+}]_i^{2.2}}$$

$$\tau_{\text{SK2}}^{-1} = -1.3 + 45.52[\text{Ca}^{2+}]_i$$

Table D19: SK2 Potassium Channel

Notation	Definition	Value
G_{SK2}	SK2 unitary conductance	2 pS
κ_{SK2}	SK2 channel density	estimated
P_{SK2_∞}	Steady state activation variable	
τ_{SK2}	Activation time constant in ms	
P_{SK2}	Channel open probability	

Small calcium-dependent potassium channel SK3- based on [24]

Channel Current (pA/pF)

$$I_{\text{SK3}} = \kappa_{\text{SK3}} G_{\text{SK3}} P_{\text{SK3}} (V - E_{\text{K}})$$

$$P_{\text{SK3}_{ss}} = \frac{[\text{Ca}^{2+}]_i^{5}}{(0.6)^{5} + [\text{Ca}^{2+}]_i^{5}}$$

$$\tau_{\text{SK3}} = 12.95 \text{ ms}$$

Table D20: SK3 Potassium Channel

Notation	Definition	Value
G_{SK3}	SK3 unitary conductance	2 pS
κ_{SK3}	SK3 channel density	estimated
P_{SK3_∞}	Steady state activation variable	
τ_{SK3}	Activation time constant in ms	
P_{SK3}	Open channel probability	

Intermediate calcium-dependent potassium channel SK4- based on [25]

Channel Current (pA/pF)

$$I_{\text{SK4}} = \kappa_{\text{SK4}} G_{\text{SK4}} P_{\text{SK4}} (V - E_{\text{K}})$$

$$\begin{aligned} P_{\text{SK4}_{ss}} &= \frac{[\text{Ca}^{2+}]_i^{3.2}}{(0.095)^{3.2} + [\text{Ca}^{2+}]_i^{3.2}} \\ \tau_{\text{SK4}} &= 5.8 \text{ ms} \end{aligned}$$

Table D21: SK4 Potassium Channel

Notation	Definition	Value
G_{SK4}	SK4 unitary conductance	11 pS
κ_{SK4}	SK4 channel density	estimated
P_{SK4_∞}	Steady state activation variable	
τ_{SK4}	Activation time constant in ms	
P_{SK4}	Open channel probability	

Na^+/Ca^{2+} exchanger NCX- based on [26]

Current (pA/pF)

$$I_{\text{NaCa}} = \kappa_{\text{NaCa}} (0.00025 e^{\frac{-0.65VF}{RT}} \frac{e^{\frac{VF}{RT}} [\text{Na}^+]_i^3 [\text{Ca}^{2+}]_o - [\text{Na}^+]_o^3 [\text{Ca}^{2+}]_i}{1 + 0.0001 [\text{Na}^+]_i^3 [\text{Ca}^{2+}]_o + [\text{Na}^+]_o^3 [\text{Ca}^{2+}]_i})$$

Plasma membrane Ca^{2+}-ATPase PMCA- based on [20]

Current (pA/pF)

$$I_{\text{PMCA}} = \kappa_{\text{PMCA}} \frac{1.15}{1 + \frac{0.0005}{[\text{Ca}^{2+}]_i}}$$

Na^{+}/K^{+} pump- based on [20]

Current (pA/pF)

$$\begin{aligned}
I_{\mathrm{NaK}} &= \kappa_{\mathrm{NaK}} I_{\mathrm{NaK_{max}}} f_{\mathrm{NaK}} I_{\mathrm{NaK,n'ai}} I_{\mathrm{NaK,ko}} \\
I_{NaK,nai} &= \frac{1}{1+(\frac{10}{[\mathrm{Na}^{+}]_i})^2} \\
I_{NaK,ko} &= \frac{1}{1+(\frac{1.5}{[\mathrm{K}^{+}]_o})} \\
f_{\mathrm{NaK}} &= \frac{1}{1+0.12e^{\frac{-0.1VF}{RT}}+0.04\sigma e^{\frac{VF}{RT}}} \\
\sigma &= 1/7(e^{\frac{[\mathrm{Na}_o^{+}]}{67.3}}-1)
\end{aligned}$$

K^{+} background current

Current (pA/pF)

$$I_{\mathrm{bgK}} = \kappa_{\mathrm{bgK}} G_{\mathrm{bgK}}(V - E_{\mathrm{K}})$$

Cl^{-} background current

Current (pA/pF)

$$I_{\mathrm{bgCl}} = \kappa_{\mathrm{bgCl}} G_{\mathrm{bgCl}}(V - E_{\mathrm{Cl}})$$

Table D22: Background,pumps and exchangers

Notation	Definition	Value
G_{bgK}	K^{+} background conductance	50 pS
G_{bgCl}	Cl^{-} background conductance	50 pS
G_{PMCA}	PMCA conductance	1 pS
G_{NaK}	Na^{+}/K^{+} conductance	1 pS
G_{bgK}	NCX conductance	1 pS
κ_{bgK}	K^{+} background density	estimated
κ_{bgCl}	Cl^{-} background density	”
κ_{PMCA}	PMCA density	”
κ_{NaK}	Na^{+}/K^{+} density	”
κ_{bgK}	NCX density	”

Table D23: General Model Notations

Notation	Definition	Value
I	the injected current	
$\varkappa_i$	the density of the conductance entity	
ψ_i	the current carried by a single molecule of type i	
g_i	the conductance of a single channel of type i	
V_i	the reversal potential of the ionic species type i	
$\boldsymbol{x}$	the gating state	
$\boldsymbol{f}$	the fully parametrized (known) kinetics	
$\boldsymbol{u}(t)$	other quantities that $\boldsymbol{f}$ could depend on	

References

[1] Y. Chan, H. van den Berg, J. Moore, S. Quenby, and A. Blanks, "Assessment of myometrial transcriptome changes associated with spontaneous human labour by high-throughput RNA-seq.," *Exp Physiol*, pp. 1–15, 2013.

[2] K. G. Klemic, C. C. Shieh, G. E. Kirsch, and S. W. Jones, "Inactivation of Kv2.1 potassium channels," *Biophys J*, vol. 74, pp. 1779–1789, 1998.

[3] A. J. Patel, M. Lazdunski, and E. Honore, "Kv2.1/Kv9.3, A novel ATP-dependent delayed-rectifier K+ channel in oxygen-sensitive Pulmonary artery myocytes," *EMBO J*, vol. 16, pp. 6615–6625, 1997.

[4] J. W. Kramer, M. A. Post, A. M. Brown, and G. E. Kirsch, "Modulation of potassium channel gating by coexpression of Kv2.1 with regulatory Kv5.1 or Kv6.1 alpha-subunits," *Am J Physiol Cell Physiol*, vol. 274, pp. C1501–C1510, 1998.

[5] B. Rudy, K. Sen, E. Vega-Saenz De Miera, D. Lau, T. Ried, and D. C. Ward, "Cloning of a human cDNA expressing a high voltage-activating, TEA-sensitive, type-A K^+ channel which maps to chromosome-1 band-p21," *J Neurosci Res*, vol. 29, pp. 401–412, 1991.

[6] H. H. Jerng and M. Covarrubias, "K^+ channel inactivation mediated by the concerted action of the cytoplasmic N- and C-terminal domains," *Biophys J*, vol. 72, pp. 163–174, 1997.

[7] T. Y. Nakamura, D. J. Pountney, S. Nandi, M. Artman, B. Rudy, and W. A. Coetzee, "Different effects of the Ca^{2+}-binding protein, KChIP1, on two Kv4 subfamily members, Kv4.1 and Kv4.2," *J Mol Cell Cardiol*, vol. 33, pp. A83–A83, 2001.

[8] E. J. Beck, M. Bowlby, W. F. An, K. J. Rhodes, and M. Covarrubias, "Remodelling inactivation gating of Kv4 channels by KChIP1, a small-molecular-weight calcium-binding protein," *J Physiol*, vol. 538, pp. 691–706, 2002.

[9] S. P. Patel, R. Parai, and D. L. Campbell, "Regulation of Kv4.3 voltage-dependent gating kinetics by KChIP2 isoforms," *J Physiol*, vol. 557, pp. 19–41, 2004.

[10] S. M. Wang, S. G. Liu, M. J. Morales, H. C. Strauss, and R. L. Rasmusson, "A quantitative analysis of the activation and inactivation kinetics of hERG expressed in Xenopus oocytes," *J Physiol*, vol. 502, pp. 45–60, 1997.

[11] M. Pusch, R. Magrassi, B. Wollnik, and F. Conti, "Activation and inactivation of homomeric KvLQT1 potassium channels," *Biophys J*, vol. 75, pp. 785–792, 1998.

[12] R. L. Schrøder, T. Jespersen, P. Christophersen, D. Strøbæk, B. S. Jensen, and S. P. Olesen, "KCNQ4 channel activation by BMS-204352 and retigabine," *Neuropharmacol*, vol. 44, pp. 553–553, 2003.

[13] R. Søgaard, T. Ljungstrøm, K. Pedersen, S. Olesen, and B. Jensen, "KCNQ4 channels expressed in mammalian cells: functional characteristics and pharmacology.," *Am J Physiol Cell Physiol*, vol. 280, pp. C859–C866, 2001.

[14] F. Doring, C. Derst, E. Wischmeyer, C. Karschin, R. Schneggenburger, J. Daut, and A. Karschin, "The epithelial inward rectifier channel Kir7.1 displays unusual K^+ permeation properties," *J Neurosci*, vol. 18, pp. 8625–8636, 1998.

[15] F. T. Horrigan and R. W. Aldrich, "Coupling between voltage sensor activation, Ca^{2+} binding and channel opening in large conductance (BK) potassium channels (vol 120, pg 267, 2002)," *J Gen Physiol*, vol. 120, pp. 599–599, 2002.

[16] L. Bao and D. H. Cox, "Gating and ionic currents reveal how the BK_{Ca} channel's Ca^{2+}sensitivity is enhanced by its beta 1 subunit," *J Gen Physiol*, vol. 126, pp. 393–412, 2005.

[17] C. J. Lingle, X. H. Zeng, J. P. Ding, and X. M. Xia, "Inactivation of BK channels mediated by the NH2 terminus of the beta 3b auxiliary subunit involves a two-step mechanism: Possible separation of binding and blockade," *J Gen Physiol*, vol. 117, pp. 583–605, 2001.

[18] B. Wang, B. S. Rothberg, and R. Brenner, "Mechanism of beta 4 subunit modulation of BK channels," *J Gen Physiol*, vol. 127, pp. 449–465, 2006.

[19] E. Perez-Reyes, L. L. Cribbs, A. Daud, A. E. Lacerda, J. Barclay, M. P. Williamson, M. Fox, M. Rees, and J. H. Lee, "Molecular characterization of a neuronal low-voltage-activated T-type calcium channel," *Nature*, vol. 391, pp. 896–900, 1998.

[20] L. Livshitz and Y. Rudy, "Uniqueness and stability of action potential models during rest, pacing, and conduction using problem-solving environment," *Biophys J*, vol. 97, pp. 1265–76, 2009.

[21] J. Arreola, J. E. Melvin, and T. Begenisich, "Activation of calcium-dependent chloride channels in rat parotid acinar cells," *J Gen Physiol*, vol. 108, pp. 35–47, 1996.

[22] M. Köhler, B. Hirschberg, C. T. Bond, J. M. Kinzie, N. V. Marrion, J. Maylie, and J. P. Adelman, "Small-conductance, calcium-activated potassium channels from mammalian brain," *Sci*, vol. 273, pp. 1709–1714, 1996.

[23] B. Hirschberg, J. Maylie, J. P. Adelman, and N. V. Marrion, "Gating of recombinant small-conductance Ca^{2+}-activated K^+ channels by calcium," *J Gen Physiol*, vol. 111, pp. 565–581, 1998.

[24] X. M. Xia, B. Fakler, A. Rivard, G. Wayman, T. Johnson-Pais, J. E. Keen, T. Ishii, B. Hirschberg, C. T. Bond, S. Lutsenko, J. Maylie, and J. P. Adelman, "Mechanism of calcium gating in small-conductance calcium-activated potassium channels," *Nature*, vol. 395, pp. 503–507, 1998.

[25] W. J. Joiner, L. Y. Wang, M. D. Tang, and L. K. Kaczmarek, "hSK4, a member of a novel subfamily of calcium-activated potassium channels," *Proc Natl Acad Sci USA*, vol. 94, pp. 11013–11018, 1997.

[26] G. M. Faber, J. Silva, L. Livshitz, and Y. Rudy, "Kinetic properties of the cardiac L-type Ca^{2+} channel and its role in myocyte electrophysiology: A theoretical investigation," *Biophys J*, vol. 92, pp. 1522–1543, 2007.
